# “Attitudes to voices”: a survey exploring the factors influencing clinicians’ intention to assess distressing voices and attitudes towards working with young people who hear voices

**DOI:** 10.3389/fpsyg.2023.1167869

**Published:** 2023-05-23

**Authors:** Aikaterini Rammou, Clio Berry, David Fowler, Mark Hayward

**Affiliations:** ^1^School of Psychology, University of Sussex, Brighton, United Kingdom; ^2^Research & Development Department, Sussex Partnership NHS Foundation Trust, Hove, United Kingdom; ^3^Brighton and Sussex Medical School, University of Sussex, Brighton, United Kingdom

**Keywords:** clinician attitudes, early intervention in mental health, psychotic experiences, voice-hearing, youth mental health, theory of planned behavior, auditory verbal hallucinations

## Abstract

**Introduction:**

Due to the general psychopathological vulnerability of young people who hear distressing voices, research has stressed the importance for clinicians to assess this experience in youth. Nonetheless, the limited literature on the topic comes from studies with clinicians in adult health services and it primarily reports that clinicians do not feel confident in systematically assessing voice-hearing and doubt the appropriateness of doing so. We applied the Theory of Planned Behavior and identified clinicians’ job attitudes, perceived behavioral control, and perceived subjective norms as putative predictors of their intent to assess voice-hearing in youth.

**Method:**

Nine hundred and ninety-six clinicians from adult mental health services, 467 from Child and Adolescent Mental Health (CAMHS) and Early Intervention in Psychosis (EIP) services and 318 primary care clinicians across the UK completed an online survey. The survey gathered data on attitudes toward working with people who hear voices, stigmatizing beliefs, and self-perceived confidence in voice-related practices (screening for, discussing and providing psychoeducation material about voice-hearing). Responses from youth mental health clinicians were compared with professionals working in adult mental health and primary care settings. This study also aimed to identify what youth mental health clinicians believe about assessing distressing voices in adolescents and how beliefs predict assessment intention.

**Results:**

Compared to other clinicians, EIP clinicians reported the most positive job attitudes toward working with young voice-hearers, the highest self-efficacy in voice-hearing practices, and similar levels of stigma. Job attitudes, perceived behavioral control and subjective norms explained a large part of the influences on clinician’s intention to assess voice-hearing across all service groups. In both CAMHS and EIP services, specific beliefs relating to the usefulness of assessing voice-hearing, and perceived social pressure from specialist mental health professionals regarding assessment practices predicted clinician intention.

**Discussion:**

Clinicians’ intention to assess distressing voices in young people was moderately high, with attitudes, subjective norms and perceived behavioral control explaining a large part of its variance. Specifically in youth mental health services, promoting a working culture that encourages opening and engaging in discussions about voice-hearing between clinicians, and with young people, and introducing supportive assessment and psychoeducation material about voice-hearing could encourage conversations about voices.

## 1. Introduction

Young people can experience shame and stigma in relation to the onset of voice-hearing experiences (Yang et al., 2015) and they may be reluctant to disclose this experience to others (Bogen-Johnston et al., 2019; Parry et al., 2020). This can result in them first asking for help with other difficulties such as peer relationships, anxiety and depression (Boydell et al., 2013; Stowkowy et al., 2013; Falkenberg et al., 2015), and rarely volunteering information on hearing voices, unless being asked directly and sensitively in a normalizing environment (Mertin and Hartwig, 2004; Kelleher et al., 2014). Moreover, young people are vulnerable to feeling that their own understanding and explanations for their voice-hearing experiences are dismissed due to the power of clinicians’ opinions and biomedical explanations (Bampton, 2012). Therefore, professionals need to have the capacity to be vigilant for and prepared to ask about hearing voices and other unusual experiences during routine assessments (Stowkowy et al., 2013), in a sensitive and normalizing way using simple and non-medical language (Sikich, 2013).

Once voice-hearing is disclosed, patients have reported a desire to discuss their experiences with mental health clinicians (Coffey and Hewitt, 2008; Bogen-Johnston et al., 2019; Griffiths et al., 2019) and receive support in managing their voice-hearing (Baker et al., 1997). Such conversations about these experiences could help patients explore their voices and potentially reduce their negative impact (Romme et al., 2009; Watkins et al., 2020). Regarding young people, a cautious-but-curious investigation of the psychopathological and psychosocial context of their voice-hearing experience is recommended (Maijer et al., 2019). This is vital, as evidence suggests that young people who report such experiences could be a target group for early intervention to improve their functional outcomes, given that psychotic-like experiences might be an early marker of later, ongoing mental distress (Lindgren et al., 2019; Carey et al., 2021). Gaining accurate and detailed information about voice-hearing may therefore facilitate clinicians in devising a helpful support plan (England, 2007). However, it appears unlikely that systematic and effective assessment of voice-hearing experiences among young people is happening consistently in routine clinical practice as clinicians appear to lack confidence in doing so (Coffey et al., 2004; Harrison et al., 2008; White et al., 2019), and can doubt the value and appropriateness of discussing voice-hearing experiences (Coffey et al., 2004; Harrison et al., 2008; White et al., 2019).

The Theory of Planned Behavior (TPB; Ajzen, 2005) is a useful model for explaining clinician behaviors (Eke et al., 2012; Levy et al., 2016; Lecomte et al., 2018). TPB proposes three main drivers of intention to perform an action: attitudes -comprising the imagined outcome of the action and how much that outcome is valued; subjective norms -comprising perceptions of what others would usually do and what they would approve or disapprove of doing, and how important that is to the person planning an action; and perceived behavioral control -comprising internal (e.g., self-efficacy for the action) and external facilitators and barriers (e.g., other duties that may need to be performed at a given time). These TPB drivers predict intention to perform the behavior, which in turn predicts actual behavior (Armitage and Conner, 2001). Thus, the TPB can explain influences on clinicians’ intention to assess voice-hearing experiences once disclosed by patients.

The attitude component of TPB can incorporate stigmatizing attitudes about voice-hearers and beliefs about the legitimacy or value of discussing voice-hearing experiences. Evidence suggests that clinicians’ attitudes are likely to be quite negative. Clinicians reportedly often struggle to discuss voice-hearing with patients, especially regarding voice content and meaning, and can feel skeptical about the value or appropriateness of such conversations (Coffey et al., 2004; Harrison et al., 2008; White et al., 2019). Mental health clinicians can hold the belief that talking about voices might do harm or cause further distress (Coffey and Hewitt, 2008; McMullan et al., 2018), which reduces the likelihood of them engaging in such conversations. More broadly, While clinicians report feeling empathic toward voice-hearers (Kramarz et al., 2020), they can feel powerless and helpless in reducing voice-related distress (McMullan et al., 2018).

The norm component of TPB could refer to clinical practice culture, which may discourage detailed discussions about voice-hearing with patients. For instance, White et al. (2019) revealed that recently qualified mental health nurses could not identify examples of colleagues having discussions with patients about their voices. This could set a workplace culture that discourages discussion about voices with patients during experiential clinical learning (Cleary et al., 2011; Wright et al., 2011).

The final component of TPB, perceived behavioral control, relates to clinicians’ perceived confidence in their ability to perform an action. Evidence suggests that clinicians report a lack of subjective understanding of voice-hearing experience and lack self-efficacy in asking detailed questions about voices (Kramarz et al., 2020). Perceived behavioral control also relates to the degree that a clinician has control over the action regarding situational factors such as time constrains (McCluskey and Vries, 2020). Moreover, lack of subjective understanding, perceived clinical risks related to commanding voices and what they might ask hearers to do (e.g., self-harm), and the diversity of voice-hearing experiences appear to be associated with professionals’ reported lack of clinical confidence (Kramarz et al., 2020).

Lastly, TPB allows for the inclusion of background factors that could influence the three main drivers of behavioral intention. These could include personal, social and informational variables that have been found to affect clinicians’ intention to discuss voices in past research (Ajzen, 2005). Evidence suggests that the quality of professional experience, (e.g., contact that disconfirms negative stereotypes or includes a common goal), is more important than the duration of work (Lauber et al., 2006; Dabby et al., 2015) in reducing stigmatizing views of mental illness (Couture and Penn, 2003; Jorm et al., 2012). Moreover, both more professional contact and having a personal relationship with someone with lived experience has been associated with less stigmatizing views of people with mental health problems in primary care clinicians and psychiatrists (Caplan et al., 2016; Sandhu et al., 2019). In terms of training in voice-hearing, general mental health professionals report little training in assessing voice-hearing (Kramarz et al., 2020; McCluskey and Vries, 2020), and even in specialist Early Intervention in Psychosis services (EIP) the training has been described as variable (Bogen-Johnston et al., 2020).

There is additionally a need to consider how assessment intention may vary for different professional groups. Although clinicians within specialist psychosis services reportedly lack self-efficacy in, and have concerns regarding, discussing voice-hearing experiences with young patients (Bogen-Johnston et al., 2020), primary care clinicians report lack of confidence in interviewing (Brunero et al., 2018) and anxiety in supporting people with mental health difficulties more generally (Roberts et al., 2013), and the least confidence in identifying and managing psychotic experiences, such as voice-hearing, in youth, compared to other mental health problems (Kehoe et al., 2020). Moreover, medical professionals have been found to hold more stigmatizing attitudes toward people with psychosis compared to mental health professionals (Hori et al., 2011; Mittal et al., 2014; Smith et al., 2017).

### 1.1. The current study

It is clear that more needs to be learnt about the responses of clinicians when a patient speaks about their distressing voice-hearing experiences (e.g., McCluskey & de Vries, 2020). The TPB offers a framework for exploring how clinician characteristics and experiences can predict intention to discuss voices. For the purposes of this study, “assessing voice-hearing” was the primary behavior of interest, referring to a detailed exploration of the experience including questions about its phenomenology (e.g., frequency, duration, content), the meaning and beliefs assigned to the voices by the patients, and the impact on their emotions and functioning. Understanding the influences on clinician intention can support the development, evaluation, and implementation of targeted training approaches.

**Figure 1:**
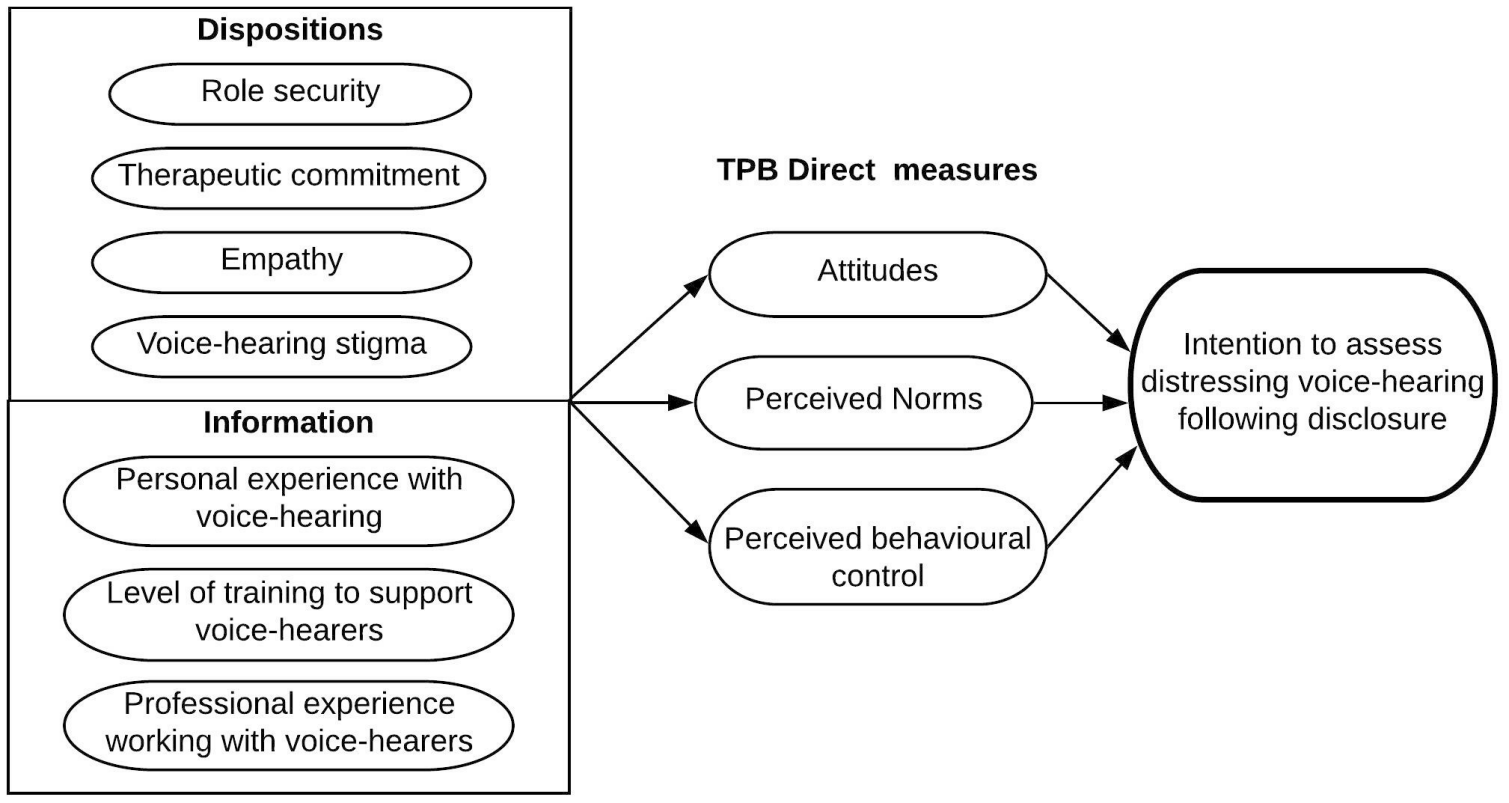
Diagrammatic representation of potential predictors of clinicians’ intention to assess distressing voice-hearing following disclosure.

With respect to the different service groups, this study aimed to explore differences in clinicians’ perceived self-efficacy in voice-hearing practice, stigma, and attitudes toward working with young people between Child and Adolescent Mental health services (CAMHS), EIP, and primary care clinicians. Adult mental health service clinicians were also sampled as a reference group, and both they, and a randomly selected half of primary care clinicians answered the questionnaire with reference to adult voice-hearers, for comparative purposes (Aim 1). This study also investigated the influence of TPB components as predictors of intention to assess distressing voice-hearing within different service groups. Based on findings about correlates to clinicians’ intention to discuss voices, the putative influence of relevant background factors on intention to assess voice-hearing was also taken into consideration. These included the dispositional factors of stigmatizing beliefs and general job attitudes toward working with patients who hear voices, and informational factors of professional and personal voice-hearing experience, perceived self-efficacy to voice-hearing practice, and past training in working with voice-hearers (see Figure 1) (Aim 2). Finally, this study aimed to identify, specifically for clinicians working with young people who hear voices, the most influential specific behavioral, normative and control beliefs on clinicians’ intention to assess distressing voices (Aim 3).

## 2. Materials and methods

### 2.1. Ethical statement

The study was sponsored by the University of Sussex, UK and received ethical approval from the Health Research Authority (Reference: 048 HAY/IRAS ID: 257355). Participants gave (electronic) informed consent for their participation before completing the self-report questionnaires online.

### 2.2. Design

This study was a between and within-group cross-sectional exploratory study using a battery of self-report questionnaires.

### 2.3. Participants and procedure

Clinicians were invited to complete an online survey by the research department of their National Health Service (NHS) Trust or the local Clinical Research Network and distribution of advertisement material. Data were collected *via* the Qualtrics online survey platform. Participants were informed that after completing and submitting the consent page of the survey that their consent was to be assumed and any data entered after that point would be recorded. Participation was anonymous and voluntary.

An *a-priori* sample size calculation for was conducted using G*Power software (Faul et al., 2009) for a multiple hierarchical linear regression model with 11 predictors of intention to assess distressing voices, indicating a minimum sample size of 262 for each service group, for an effect size of *f* ^2^ = 0.10, a = 0.05 and power of 0.95. Participants had to be clinicians working in an NHS service; Child and Adolescent Mental Health Services (CAMHS), Early Intervention in Psychosis (EIP) Services and/or adult mental health services, or in primary care services. Nine hundred and ninety-six clinicians from adult mental health services, 467 from CAMHS and EIP, working in 27 NHS mental health Trusts took part in the study. In primary care services, 158 clinicians completed the survey asking about adult patients and 160 the survey asking about young patients (12–18 years of age), working in 32 Integrated Care Boards across the UK.

To allow for comparison between predictors of intention to assess distressing voice-hearing depending on the age of patients that clinicians typically work with within their respective services, CAMHS and EIP clinicians completed a survey with reference to patients who were 12–18 years of age, whereas Adult mental health service clinicians were asked about patients aged 19 years and over. Primary care clinicians were randomized so that half of them completed the survey with reference to patients 12–18 years of age and the remaining half about patients 19 years or over. Participant demographic and professional background characteristics appear in Table 1.

**Table 1:** Sample characteristics for all service groups (*N* = 1751).

Sample characteristic	Adult Mental health (*N* = 996)	EIP (*N* = 253)	CAMHS (*N* = 214)	Primary Care (Adult service user version) (*N* = 158)	Primary Care (Young service user version) (*N* = 160)
***M* (Min–Max; SD)**					
Age (years)	40.75 (18–72; 11.71)	39.97 (19–68;10.60)	38.26 (20–71; 10.49)	44.65 (25–67; 9.53)	45.47 (23–69; 9.20)
Experience in current profession (years)	11.22 (0–49; 10.54)	9.7 (0–35; 9.24)	8.92 (0–39; 8.88)	14.59 (0–40; 10.58)	14.31 (0–44; 10.48)
Experience in mental health services	12.93 (0–49; 10.29)	13.48 (0–35; 9.36)	10.41 (0–40;8.54)	1.26 (0–25; 4.34)	1.49 (0–44; 5.98)
Experience in young people mental health services	–	4.99 (0–25; 5.10)	6.85 (0–33; 6.71)	2.25 (0–11; 3.79)^a^	2.61 (0–22; 6.47)^a^
Experience in adult people mental health services	11.26 (0–48; 9.68)	–		2.47 (0–25; 5.26)^b^	2.91 (0–36; 6.65)^b^
***N* (Valid %)**					
Gender					
Male	278 (28.78)	65 (25.69)	45 (21.03)	46 (29.11)	61 (38.13)
Female	671 (69.46)	184 (72.73)	168 (78.50)	112 (70.89)	98 (61.25)
Another gender identity	4 (0.41)	2 (0.79)	1 (0.47)	0	0
Prefer not to say	13 (1.35)	2 (0.79)	0	0	1 (0.63)
Ethnicity					
White British	701 (72.57)	215 (84.98)	169 (79.34)	115 (72.78)	107 (66.88)
White Other	11 (11.59)	16 (6.32)	22 (10.33)	13 (8.23)	6 (3.75)
Asian/Asian British	47 (4.87)	9 (3.56)	10 (4.69)	22 (13.92)	32 (20)
Black/African/Caribbean/Black British	46 (4.76)	9 (3.56)	3 (1.41)	2 (1.27)	4 (2.50)
Mixed Ethnicity	28 (2.90)	3 (1.19)	6 (2.82)	2 (1.27)	6 (3.75)
Another ethnic group	12 (1.14)	1 (0.40)	2 (0.94)	3 (1.90)	2 (1. 25)
Prefer not to say	20 (2.07)	0	1 (0.47)	1 (0.63)	3 (1.88)
Nationality					
British	814 (84.27)	234 (92.49)	189 (88.32)	140 (88.61)	142 (88.75)
Old EU	59 (6.11)	8 (3.16)	13 (6.07)	5 (3.16)	4 (2.50)
New EU	17 (1.76)	0	4 (1.87)	2 (1.27)	0
Another nationality	62 (6.42)	11 (4.35)	7 (3.27)	10 (17.24)	11 (18.33)
Prefer not to say	14 (1.45)	0	1 (0.47)	1 (0.63)	3 (1.88)
Secondary care mental health service type					
EIP		253 (100)	0	–	–
Looked After CAMHS	–	–	7 (3.27)	–	–
Community CAMHS/AMHS (Tier 3)	437 (45.24)	–	141 (65.89)	–	–
Community CAMHS (Tier 2)	–	NA	16 (7.48)	–	–
Community CAMHS (Neuro behavioral Clinic)	–	NA	2 (0.93)	–	–
Inpatient	248 (25.67)	NA	30 (14.02)	-	–
Specialist service, e.g., Assertive outreach	164 (16.98)	–	–	–	–
Youth offending service	–	NA	2 (0.93)	–	–
Another type of service	117 (12.11)	NA	16 (7.48)	–	–
Primary care team type					
General Practice	–	–	–	145 (92.36)	150 (94.34)
Primary mental health	–	–	–	2 (1.27)	1 (0.63)
IAPT	–	–	–	8 (5.10)	6 (3.77)
Another type of service				2 (1.27)	2 (1.26)
Professional background					
Psychological wellbeing practitioner	12 (12.42)	0	2 (0.93)	2 (1.27)	4 (2.5)
Psychiatrist	109 (11.28)	14 (5.53)	21 (9.81)	1 (0.63)	0
Mental health/Senior mental health nurse	318 (32.92)	86 (33.99)	56 (0.26)	0	0
Clinical psychologist	72 (7.45)	27 (10.67)	31 (14.49)	1 (0.63)	0
Counseling psychologist	14 (1.45)	4 (1.58)	0	–	0
Counselor	8 (0.83)	3 (1.19)	2 (0.93)	–	0
Art therapist	9 (0.93)	0	7 (3.27)	–	0
Cognitive behavioral therapist	23 (2.38)	27 (10.67)	15 (7.01)	2 (1.27)	1 (0.63)
Psychotherapist	16 (1.66)	2 (0.79)	18 (8.41)	4 (2.53)	1 (0.63)
Social Worker	53 (5.49)	30 (11.86)	21 (9.81)	–	–
Assistant psychologist	50 (5.18)	15 (5.93)	24 (11.21)	–	–
Occupational therapist	56 (5.80)	24 (9.49)	5 (2.34)	–	–
Support, time, and recovery worker	3 (0.31)	0	0	–	–
Nursing Trainee	19 (1.97)	1 (0.40)	1 (0.47)	0	0
Clinical psychologist trainee	20 (2.07)	3 (1.19)	4 (1.87)	–	–
Healthcare assistant/ Support worker	123 (12.73)	20 (7.91)	23 (10.7)	9 +	8 (5)
Student other	39 (4.04)	7 (2.77)	11 (5.14)	3 (1.90)	–
Research	24 (2.48)	3 (1.19)	0	–	0
Other	84 (8.70)	23 (9.09)	1 (0.5)	8 (5.06)	9 (5.63)
Practice nurse/Nurse practitioner	–	–	–	24 (15.19)	37 (23.13)
General practitioner	–	–	–	103 (65.19)	95 (59.38)
Practice paramedic	–	–	–	3 (1.90)	7 (4.38)
Had any work experience in mental health	–	–	–	66 (41.77)	71 (44.38)

a Included only clinicians with experience working in young people mental health services, *N* = 18 for primary care clinicians who completed the young patient survey, *N* = 16 for primary care clinicians who completed the adult patient survey.

b Included only clinicians with experience working in adult mental health services, *N* = 66 for primary care clinicians who completed the young patient survey, *N* = 66 for primary care clinicians who completed the adult patient survey. *N* = Valid % represents percentage of participants with the available data; EIP, Early Intervention in Psychosis services; CAMHS, Child and Adolescent Mental Health services; IAPT, Improving Access to Psychological Therapies services; AMHS, Adult Mental Health Services. Old EU refers to Austrian, Belgian, Danish, Dutch, Finnish, French, German, Greek, Irish, Italian, Luxembourger, Portuguese, Spanish, and Swedish nationalities. New EU refers to Bulgarian, Croatian, Cypriot, Czech, Estonian, Hungarian, Latvian, Lithuanian, Maltese, Polish, Romanian, Slovakian, and Slovenian nationalities.

### 2.4. Measures

#### 2.4.1. Demographic and professional background.

Demographic and professional background data relating to profession, years of experience in mental health services in general, and in young people’s services more specifically were captured. Clinicians were also asked to indicate the total number of voice-hearers they had worked with, frequency of contact with patients with distressing voices, personal experience with voice-hearing, level, and type of training to support patients who hear voices, and their perceived need and willingness to receive further training on supporting and assessing distressed voice-hearers.

#### 2.4.2. Voice-hearing practice self-efficacy.

A self-efficacy questionnaire was constructed following recommendations by Bandura (2006), with three items aiming to measure clinicians’ self-perceived capability for three different tasks: (1) ask a patient if they hear voices; (2) discuss voice-hearing experiences with a patient who hears distressing voices; and (3) provide useful information to a patient who hears distressing voices. The items were rated on a 100-point scale, from 0 = “Cannot do” to 100 = “Highly certain can do.”

#### 2.4.3. Theory of planned behavior (TPB).

##### 2.4.3.1. Direct measures.

A TPB measure was constructed to capture clinicians’ intention to assess distressing voice-hearing following patient disclosure, following author guidelines (Fishbein and Ajzen, 2010) and additional researcher recommendations (Francis et al., 2004). A definition of the term “assessing” was provided; “asking a service user a series of open-ended questions to get detailed information about their experience”. The measure included items relating to the direct predictors of intention to assess voice-hearing, namely attitudes (*n* = 7 items, e.g., “When a young person discloses hearing distressing voices to me, assessing their voice-hearing experiences would be…,” with a response scale from “Very harmful” to “Very beneficial”), subjective norms (*n* = 5; e.g., “When a young person discloses hearing distressing voices to me, most people who are important to me professionally would approve of my assessing their voice-hearing experiences” with a response scale from “Very strongly disagree” to “Very strongly agree”), and perceived behavioral control (*n* = 6; e.g., “When a young person discloses hearing distressing voices to me, I am confident that I will assess their voice-hearing experiences”; with a response scale from “Very strongly disagree” to “Very strongly agree”). Three items captured behavioral intention (e.g., “When a young person discloses hearing voices to me, I intend to assess their voice-hearing experiences from now on”; with a response scale from “Very strongly disagree” to “Very strongly agree”).

Responses were recorded on a 7-point Likert scale, with higher scores representing more positive attitudes, approving subjective norms, and higher perceived behavior control.

The mean of item scores was calculated to provide an overall construct score. The TPB subscales for all predictor factors showed good internal consistency in all service groups, αs > 0.79. Specifically, for the attitudes subscale, the internal consistency estimates were α = 0.82 for the adult mental health, α = 0.82 for youth mental health (EIP and CAMHS), α = 0.90 for adult primary care and α = 0.87 for primary care for young people. The internal consistency estimates for the subjective norms subscale were α = 0.81 for the adult mental health, α = 0.79 for youth mental health (EIP and CAMHS), α = 0.90 for adult primary care and α = 0.85 for primary care for young people. The internal consistency estimates for the perceived behavioral control scale were α = 0.85 for the adult mental health, α = 0.85 for youth mental health (EIP and CAMHS), α = 0.89 for adult primary care and α = 0.85 for primary care for young people. For the intention subscale, the internal consistency was α = 0.93 for the adult mental health, α = 0.94 for youth mental health (EIP and CAMHS), α = 0.96 for adult primary care and α = 0.98 for primary care for young people.

##### 2.4.3.2. Indirect measures.

The TPB questionnaire for the EIP and CAMHS clinicians additionally included indirect, belief-based predictors in the form of 30 specific belief items associated with forming attitudes, subjective norms, and perceived behavioral control regarding assessing voice-hearing and the outcome evaluation for each belief (Ajzen, 1991; Supplementary Table S1). Indirect measures were calculated by multiplying the individual belief with its corresponding outcome evaluation and then summing the products for each TPB predictor construct. Higher scores indicate that a clinician is in favor of, experiences social pressure to, and feels in control of assessing voice-hearing. Further details on the development and scoring of the indirect measures can be found in the Supplementary Method Information S1 and Supplementary Table S2. The final TPB questionnaire for the CAMHS and EIP clinicians had 76 items, 53 of which related to the indirect TPB measures.

#### 2.4.4. Attitudes relating to working with people with distressing voices.

To measure clinicians’ attitudes relating to working with people with distressing voices, a 35-item modified version (McLeod et al., 2002) of the Alcohol and Alcohol Problems Perception Questionnaire (AAPPQ; Cartwright, 1980) was used. The original AAPPQ had been designed to capture therapists’ attitudes toward working with patients who abuse alcohol, and it has been modified (McLeod et al., 2002; Berry and Greenwood, 2016) to capture attitudes of mental health professionals to working with people with psychosis. For the purpose of this study, the questionnaire items were amended to refer specifically to voice-hearing. Items were scored on a 7-point Likert scale, with higher scores reflecting more positive attitudes. The following mean subscale scores were calculated, all of which had acceptable internal consistency: role security (before item removal) α = 0.87 for adult mental health, α = 0.89 for youth mental health, α = 0.89 for adult primary care, α = 0.91 for young people primary care; role security after two items were removed (“I feel I have the right to ask a client for any information that is relevant to their problems with distressing voice-hearing” and “I feel I have the right to ask clients questions about their distressing voice-hearing when necessary”) α = 0.92 for adult mental health, α = 0.90 for youth mental health, α = 0.90 for adult primary care, α = 0.91 for young people primary care after two items being removed); therapeutic commitment (before item removal) α = 0.87 for adult mental health, α = 0.90 for youth mental health, α = 0.83 for adult primary care, α = 0.86 for young people primary care; therapeutic commitment after one item was removed (“I wish there was more respect to staff who work with service users who hear distressing voices”) α = 0.88 for adult mental health, α = 0.91 for youth mental health, α = 0.85 for adult primary care, α = 0.87 for young people primary care; and empathy (before item removal) α = 0.69 for adult mental health, α = 0.72 for youth mental health, α = 0.69 for adult primary care, α = 0.66 for young people primary care; and empathy after one item was removed (“I find it difficult to have empathy for service users’ experience of hearing distressing voices”) α = 0.75 for adult mental health, α = 0.77 for youth mental health, α = 0.71 for adult primary care, α = 0.66 for young people primary care.

#### 2.4.5. Stigma toward voice-hearing.

The Attribution Questionnaire-9 [AQ-9; adapted from Corrigan et al., (2014)] was used to capture stigma toward people who experience voice-hearing. The questionnaire presents a vignette portraying a male patient with a diagnosis of schizophrenia. As voice-hearing can be present in different diagnoses (Larøi, 2012; Maijer et al., 2017), the diagnosis was removed and to minimize bias related to patient gender, the language was amended to be gender-neutral. Alongside the vignette, nine questions were used to assess responsibility (blame, pity, danger, and help) and dangerousness (danger, fear, avoidance, coercion, and institutionalization). Each response is scored on a 9-point Likert scale, from 1 (Not at all) to 9 (Very much). Clinician stigma was estimated as the total score of all items, with higher scores representing more stigmatizing attitudes. Corrigan et al. (2014) found AQ-9 internal consistency and test–retest reliability for mental health clinicians to be 0.71 and 0.87, respectively. The internal consistency estimates in this study were α = 0.69 for adult mental health, α = 0.57 for youth mental health, α = 0.68 for adult primary care and α = 0.55 for young people primary care. To improve the internal consistency of AQ-9, two items were removed from the total score estimation (“I would feel pity for Sam,” “How likely is it that you would help Sam?”), resulting in Cronbach’s alpha of α = 0.75 for adult mental health, α = 0.70 for youth mental health, α = 0.75 for adult primary care and α = 0.69 for young people primary care.

### 2.5. Data analysis

Participants’ responses to the survey were exported to SPSS Version 25 (IBM Corp., 2017). Welch’s t-tests and Pearson’s chi-square tests were used to investigate whether data missingness was related to any demographic or background variables either within each service group or in the whole sample. A Bonferroni-corrected value of p accounted for multiple comparisons (*p* = 0.005).

To explore differences in clinicians’ perceived self-efficacy in voice-hearing practice, stigma, and attitudes toward working with patients who hear voices (aim 1), two one-way MANOVAs were used to identify any differences in voice-hearing practice self-efficacy (first model), and in attitudes toward working with people who hear voices and stigma (second model) between clinicians working in CAMHS, EIP, Adult Mental health and Primary Care services. *Post hoc* tests with Bonferroni corrections were used in line with recommendations from Field (2017). ANOVAs were conducted to investigate the effect of service group on each outcome variable, using Browne-Forsythe F robust test with a Bonferroni corrected value of *p* = 0.007, and Hedges’ g effect size corrected for unequal sample sizes (Hedges and Olkin, 1985). Due to univariate normality tests showing distributional issues, Pillai’s trace test was selected (Field, 2017).

To explore the influence of TPB direct measures and other background factors as predictors to assess distressing voice hearing following patient disclosure within different service groups (aim 2), a hierarchical multiple regression analysis was performed. The multiple regression model was conducted separately for the adult mental health, CAMHS, EIP and primary care service groups and for the overall sample. The hierarchy of entry for predictors was as follows: first direct TPB measures of attitude, subjective norms and perceived behavioral control were entered; secondly, all remaining explanatory variables (m-AAPPQ role security, therapeutic commitment, empathy, and total AQ-9 stigma scores) were added (Figure 1); finally, dummy variables for personal experience with voice-hearing (Yes/No), specific training in working with voice-hearers (Formal training vs. No formal training but considerable clinical experience vs. No training and/or minimal experience) and professional experience of working with voice-hearers (having worked with more vs. less than 10 voice-hearers) were added. When testing the model with the total sample, dummy variables representing service group (EIP, CAMHS, primary care groups versus adult mental health clinicians being the reference category) were added in the first block to investigate whether the type of service significantly contributes to intention to assess distressing voices in patients. Effect sizes for individual predictors were calculated using Cohen’s *f* ^2^ (Cohen, 1988). To identify specific indirect TPB behavioral, normative, control beliefs that exerted the greatest influence on clinicians’ intention to assess distressing voices, the sample was dichotomized based on no/low versus. moderate/high intention (Francis et al., 2004). Two binary logistic regression models were run separately within the CAMHS and the EIP clinicians. The models were build based on the principle of parsimony, including only predictors that improved the model (Field, 2017).

Data assumptions underlying the MANOVAs (aim 1), hierarchical linear (aim 2) and logistic regression models (aim 3) were tested (Field, 2017) within each and in the overall sample. All main analyses were conducted with and without potential outlier cases. Differences in results following the removal of outliers are reported where they occurred.

To mitigate any multivariate normality deviations, confidence intervals and significance values estimation for both type of regressions were based on the Bootstrapped results (BCa 95%CI and *N* = 2000 samples). For Aim 2, robust regressions were also run as a sensitivity analysis to ensure regression coefficients were not biased due to any homoscedasticity issues. The missing values analysis for the variables used in all groups indicated that the highest rate of missing cases was for the stigma AQ-9 scale in all service groups, ranging from 8.9% (*N* = 14) in primary care clinicians who responded the survey for adult patients to 34.1% (*N* = 73) in CAMHS clinicians (see Supplementary Analysis Information S1, Supplementary Tables S3, S4 for further details). Missing values analysis for the additional 30 indirect TPB belief items completed by the EIP clinicians revealed differences between completers and non-completers in all control belief items (*p*s <. 005). Completers of the items were older and had more experience working in mental health services than non-completers (*p*s = or < 0.005). There were no significant differences between completers and non-completers of indirect TPB belief items in CAMHS clinicians (*p*s > 0.005). Pairwise deletion of cases was selected, using all available cases in each analysis. Descriptive statistics of the study variables are summarized in Supplementary Tables S5, S6.

## 3. Results

### 3.1. Aim 1: service group differences in voice-hearing practice self-efficacy, stigma, and attitudes toward working with people who hear voices

Using Pillai’s trace, there was a significant effect of type of service on clinicians’ voice-hearing practice self-efficacy, *V* = 0.30, *F* (12,4,908) = 45.18, *p* < 0.001, partial *η*^2^ = 0.10 (Table 2).

**Table 2:** ANOVAs results for the voice-hearing practice self-efficacy scales, attitudes toward working with patients who hear voices (m-AAPPQ subscales) and stigma (AQ-9) by clinicians’ type of service.

Outcome variable	Predictor	Sum of squares	Mean square	*df*	*F*	*p*	Partial *η*^2^
Self-efficacy to ask a patient if they hear voices	Intercept	7,633,455	7,633,455	1	21297.4	<0.001	–
	Type of service	102200.95	25550.24	4	48.11	<0.001	0.15
	Error	586379.78	358.42	1,636		–	–
Self-efficacy to discuss voice-hearing with a patient	Intercept	6699727.2	6699727.2	1	16318.3	<0.001	–
	Type of service	164706.68	41176.67	4	68.71	<0.001	0.20
	Error	671683.41	410.56	1,636	–	–	–
Self-efficacy to provide useful information about voice-hearing to a patient	Intercept	3,444,394	3,444,394	1	5117.85	<0.001	–
	Type of service	372821.37	93205.34	4	143.29	<0.001	0.25
	Error	1101053.8	673.02	1,636	-	–	–
AQ-9 Stigma	Intercept	1077.95	1077.96	1	41600.9	<0.001	–
	Type of service	2.49	0.62	4	23.73	<0.001	0.07
	Error	35.86	0.03	1,384	–	–	–
m-AAPPQ Therapeutic Commitment	Intercept	2503.78	2503.78	1	43150.4	<0.001	–
	Type of service	31.91	7.98	4	150.33	<0.001	0.28
	Error	80.31	0.06	–	–	–	–
m-AAPPQ Role Security	Intercept	2945.26	2945.26	1	33369.7	<0.001	
	Type of service	36.72	9.18	4	109.1	<0.001	0.23
	Error	122.15	0.09	1,384	–	–	–
m-AAPPQ Empathy	Intercept	16905.59	16905.59	1	11716.8	<0.001	
	Type of service	118.78	29.69	4	22.48	<0.001	0.06
	Error	1996.91	1.44	1,384	–	–	–

The *F* value for the main model is based on the Brown-Forsythe robust *F*-test value. The transformed AQ-9, m-AAPPQ Therapeutic commitment and m-AAPPQ Role Security variables have been used for the models. m-AAPPQ, modified Alcohol and Alcohol Problems Perception Questionnaire; AQ-9, Attribution Questionnaire-9.

*Post hoc* Games Howell tests showed that adult mental health clinicians did not significantly differ from CAMHS clinicians in self-efficacy scores in asking a patient if they hear voices and in discussing voice-hearing with a patient distressed by voices (*p* = 0.801 and *p* = 0.128 respectively). However, both adult and CAMHS clinicians had higher scores than primary care clinicians, irrespective of the target patient age group (*p*s < 0.001), with Hedge’s g ranging from 0.68 to 1.27. There were no differences among primary care clinicians based on the target patient age group being adult or young people (*p* = 0.919 and *p* = 0.979 respectively). Self-efficacy to provide useful information to patients with distressing voices showed similar differences between the five service groups, although CAMHS and adult mental health services clinicians seemed to be different, with adult mental health clinicians having higher scores from CAMHS clinicians (*p* = 0.047, *g* = 0.20), although this result was not robust to Bonferroni correction. Again, the scores of the primary care clinicians did not differ significantly according to target patient age (*p* = 0.866). EIP clinicians had significantly higher scores in all self-efficacy items compared to all other groups (*p* < 0.001), with effect sizes ranging from *g* = 0.31 to 2.17.

Pillai’s trace also indicated a significant effect of service type on clinician stigma, therapeutic commitment, role security and empathy scores, *V* = 0.30, *F* (12, 4,194) = 38.92, *p* < 0.001, partial *η*^2^ = 0.10 (Table 2).

*Post hoc* Games Howell tests showed that adult mental health, CAMHS, and EIP service groups did not differ significantly from each other in stigma (*p*s > 0.05) but reported significantly reduced stigma compared to primary care clinicians (*p* < 0.001, *g* = 0.61–0.81). Primary care practitioner stigma scores did not differ with reference to adult versus young person target patients (*p* = 0.999). Therapeutic commitment scores were significantly higher for EIP clinicians compared to all other service groups (*p*s < 0.001, *g* = 0.52–2.04), and higher for adult mental health compared to CAMHS clinicians (*p* = 0.022, *g* = 0. 28). Primary care clinicians scored lower on therapeutic commitment compared to all mental health clinicians (*p* < 0.001, *g* = 1.17–2.06) but did not differ depending on target patient age (*p* = 0.767). EIP clinicians reported greater role security scores compared to all other clinicians (*p*s < 0.001, *g* = 0.57–1.94). There was no difference in role security between CAMHS and adult clinicians (*p* = 0.130). The primary care service groups scored lower compared to all mental health clinicians (*p* < 0.001, *g* = 0.88–1.94), and this score did not differ depending on target patient age (*p* = 0.418). EIP clinicians reported greater empathy compared to all other service groups (adult mental health and primary care clinicians, *p* < 0.001, *g* = 0.033 and *g* = 0.80–0.95; CAMHS, *p* = 0.003, *g* = 0.40). Adult mental health clinicians reported significantly greater empathy than primary care clinicians (*p*s < 0.001, *g* = 0.41–0.53) but not compared to CAMHS clinicians (*p* = 0.931). Primary care clinicians scored lower on empathy compared to all mental health clinicians (*p* < 0.001, *g* = 0.41–0.95), however, when the target patient age was 12–18 years, there was no difference in reported empathy among primary care compared to CAMHS clinicians (*p* = 0.032).

### 3.2. Aim 2: Predictors of intention To assess distressing voice-hearing across different service groups

For adult mental health clinicians, intention to assess distressing voice-hearing was significantly predicted by more positive TPB attitudes toward doing so, *f* ^2^ = 0.02, and subjective norms, *f* ^2^ = 0.03, greater perceived behavioral control, *f* ^2^ = 0.03, greater therapeutic commitment, *f* ^2^ = 0.004, and reduced empathy, *f* ^2^ = 0.01. The final model was a significant fit to the data. The predictors explained 52% of the intention to assess voice-hearing, with TPB measures, namely attitudes, subjective norms and perceived behavioral control, accounting for 98.1% of that (Table 3). In CAMHS clinicians, more positive TPB subjective norms, *f* ^2^ = 0.01, lower therapeutic commitment, *f* ^2^ = 0.03, greater role security, *f* ^2^ = 0.02, and greater empathy, *f* ^2^ = 0.02, were significant predictors of intention. The final regression model significantly fitted the data. The model explained 60% of the variance in intention, of which subjective norms explained 91.7% (Table 4). In EIP clinicians (Table 5), both TPB attitudes, *f* ^2^ = 0.01, and subjective norms, *f* ^2^ = 0.02, predicted intention to assess voices, but presence of either self-reported formal training and/or considerable experience working with voice-hearers, *f* ^2^ = 0.02, had a negative relationship with intention to assess voice-hearing. Again, the final model significantly predicted clinicians’ intention, with most of the variance in clinicians’ intention explained by the TPB measures.

**Table 3:** Linear model of predictors of TPB intention to assess distressing voice-hearing after disclosure of the experience in adult mental health clinicians (*N* = 966).

Variable	*b*	SE B	*β*	*p*	95%CI for b	*R*^2^	Adjusted^a^ R^2^	Δ*R*^2^	*F*(df)	*t*
Step 1						0.51	0.51	0.51	266.33 (3,777), p < 0.001	
Constant	0.63	0.2	–	0.008	(0.17, 1.10)					3.15
TPB Attitudes	0.36	0.06	0.27	<0.001	(0.22, 0.50)					6.58
TPB Subjective Norms	0.27	0.04	0.24	<0.001	(0.17, 0.37)					6.81
TPB Perceived behavioral control	0.33	0.05	0.29	<0.001	(0.19, 0.48)					6.55
Step 2						0.52	0.52	0.01	119. 40 (7, 773), p < 0.001	
Constant	0.38	0.28	-	0.21	(−0.21, 0.95)					1.35
TPB Attitudes	0.38	0.06	0.29	<0.001	(0.23, 0.51)					6.25
TPB Subjective Norms	0.26	0.04	0.23	<0.001	(0.17, 0.38)					6.8
TPB Perceived behavioral control	0.38	0.05	0.33	<0.001	(0.23, 0.51)					7.16
**m-AAPPQ – Therapeutic commitment**	0.16	0.07	0.10	0.03	(0.03, 0.31)					2.46
**m-AAPPQ – Role security**	−0.1	0.05	−0.10	0.04	(−0.21, −0.0004)					−2.2
**m-AAPPQ – Empathy**	−0.1	0.03	−0.10	0.001	(−0.15, −0.04)					−3.47
**AQ-9 Stigma**	0.01	0.01	0.03	0.24	(−0.004, 0.02)					1.17
Step 3						0.52	0.52	0.001	75.82 (11, 769), p < 0.001	
Constant	0.38	0.29	-	0.21	(−0.23, 1.01)					1.32
TPB Attitudes	0.38	0.06	0.29	<0.001	(0.23, 0.51)					6.28
TPB Subjective Norms	0.26	0.04	0.22	<0.001	(0.17, 0.37)					6.65
TPB Perceived behavioral control	0.38	0.05	0.33	<0.001	(0.23, 0.51)					7.17
m-AAPPQ – Therapeutic commitment	0.16	0.07	0.10	0.03	(0.03, 0.31)					2.47
m-AAPPQ – Role security	−0.1	0.06	−0.08	0.14	(−0.19, 0.03)					−1.74
m-AAPPQ – Empathy	−0.1	0.03	−0.10	<0.001	(−0.14, −0.03)					−3.34
AQ-9 Stigma	0.01	0.01	0.03	0.30	(−0.01, 0.02)					1.08
**Worked with 10 or more voice-hearers**	−0.1	0.09	−0.02	0.43	(−0.25, 0.12)					−0.84
**Personal experience with voice-hearing**	0	0.07	−0.01	0.49	(−0.21, 0.10)					−0.54
**Formal training on voice-hearing** ^**a**^	0	0.14	−0.02	0.58	(−0.42, 0.27)					−0.28
**No formal training but considerable experience on voice-hearing** ^**a**^	0	0.14	−0.01	0.73	(−0.37,0.28)					−0.1

a The reference category for training on helping voice-hearers the group without formal training nor considerable experience. *R*^2^, proportion of the variance explained; *F*, F-ratio; *t*, *t*-test; CI, confidence intervals. TPB, Theory of Planned Behavior; m-AAPPQ, modified Alcohol and Alcohol Problems Perception Questionnaire; AQ-9, Attribution Questionnaire-9. 95% bias corrected and accelerated confidence intervals reported in parentheses. Confidence intervals and standard errors are based on *N* = 2000 bootstrapped samples.

Variables in bold font represent the added variables in each block of the hierarchical regression.

**Table 4:** Linear model of predictors of TPB intention to assess distressing voice-hearing after disclosure of the experience in CAMHS clinicians (*N* = 214).

Variable	*b*	SE B	*β*	*p*	95%CI for b	*R*^2^	Adjusted^a^ R^2^	Δ*R*^2^	*F*(*df*)	*t*
Step 1						0.55	0.54	0.55	54.75 (3, 136), *p* < 0.001	
Constant	1.4	0.4	–	0.01	(0.44, 2.62)					3.47
TPB Attitudes	0.28	0.11	0.24	0.08	(−0.02, 0.58)					2.55
TPB Subjective Norms	0.33	0.07	0.32	<0.001	(0.18, 0.49)					4.38
TPB Perceived behavioral control	0.26	0.09	0.29	0.05	(−0.01, 0.50)					2.97
Step 2						0.60	0.58	0.06	28.83 (7,132), *p* < 0.001	
Constant	2.06	0.53		<0.001	(0.85, 3.21)					3.87
TPB Attitudes	0.3	0.12	0.25	0.04	(0.04, 0.52)					2.51
TPB Subjective Norms	0.31	0.08	0.3	<0.001	(0.17, 0.48)					4.1
TPB Perceived behavioral control	0.22	0.1	0.24	0.17	(−0.09, 0.44)					2.24
**m-AAPPQ-Therapeutic commitment**	−0.45	0.13	−0.33	<0.001	(−0.71, −0.15)					−3.5
**m-AAPPQ-Role security**	0.27	0.1	0.28	0.02	(0.03, 0.53)					2.57
**m-AAPPQ-Empathy**	0.13	0.06	0.15	0.02	(0.03, 0.23)					2.22
**AQ-9 Stigma**	0.01	0.01	0.05	0.02	(−0.02, 0.04)					0.81
Step 3						0.60	0.57	0.001	17.704 (11,128), *p* < 0.001	
Constant	2.1	0.55		<0.001	(0.84, 3.42)					3.79
TPB Attitudes	0.29	0.12	0.24	0.08	(−0.10, 0.52)					2.35
TPB Subjective Norms	0.31	0.08	0.3	<0.001	(0.16, 0.51)					3.92
TPB Perceived behavioral control	0.22	0.1	0.24	0.17	(−0.09, 0.44)					2.2
m-AAPPQ – Therapeutic commitment	−0.45	0.13	−0.33	<0.001	(−0.73, −0.16)					−3.39
m-AAPPQ – Role security	0.26	0.12	0.27	0.04	(0.01, 0.56)					2.25
m-AAPPQ – Empathy	0.13	0.06	0.15	0.03	(0.02, 0.24)					2.2
AQ-9 Stigma	0.01	0.01	0.05	0.52	(−0.02, 0.04)					0.8
**Worked with 10 or more voice-hearers**	0.07	0.18	0.03	0.49	(−0.26, 0.60)					0.4
**Personal experience with voice-hearing**	−0.01	0.13	−0.01	0.86	(−0.23, 0.26)					−0.11
**Formal training on voice-hearing** ^**a**^	−0.03	0.22	−0.01	0.72	(−0.56, 0.38)					−0.12
**No formal training but considerable experience on voice-hearing** ^**a**^	−0.02	0.21	−0.01	0.71	(−0.56, 0.35)					−0.11

a The reference category for training on helping voice-hearers the group without formal training nor considerable experience. *R*^2^, proportion of the variance explained; F, F-ratio; *t*, *t*-test; CI, confidence intervals. TPB = Theory of Planned Behavior; m-AAPPQ = modified Alcohol and Alcohol Problems Perception Questionnaire; AQ-9 = Attribution Questionnaire-9. 95% bias corrected and accelerated confidence intervals reported in parentheses. Confidence intervals and standard errors are based on 2000 bootstrapped samples. Variables in bold font represent the added variables in each block of the hierarchical regression.

**Table 5:** Linear model of predictors of TPB intention to assess distressing voice-hearing after disclosure of the experience in EIP clinicians (*N* = 253).

Variable	*b*	*SE B*	*β*	*p*	*95%CI for b*	*R*^2^	*Adjusted* ^a^	*ΔR*^2^	*F(df)*	*t*
							*R*^2^			
Step 1						0.52	0.51	0.52	65.55 (3,184), *p* < 0.001	
Constant	1.53	0.35		<0.001	(0.79, 2.67)					4.36
TPB Attitudes	0.54	0.09	0.51	<0.001	(0.30, 0.77)					5.98
TPB Subjective Norms	0.16	0.06	0.16	0.02	(0.03,0.31)					2.43
TPB Perceived behavioral control	0.13	0.09	0.13	0.39	(−0.13, 0.28)					1.5
Step 2						0.52	0.51	0.01	28.27 (7,180), *p* < 0.001	
Constant	1.72	0.5		<0.001	(0.80, 2.91)					3.46
TPB Attitudes	0.59	0.1	0.55	<0.001	(0.32, 0.82)					6.05
TPB Subjective Norms	0.16	0.07	0.16	0.03	(0.03, 0.32)					2.47
TPB Perceived behavioral control	0.16	0.09	0.15	0.29	(−0.11, 0.30)					1.73
**m-AAPPQ – Therapeutic commitment**	−0.11	0.13	−0.09	0.67	(−0.30, 0.22)					−0.86
**m-AAPPQ – Role security**	0.02	0.1	0.02	0.94	(−0.23, 0.21)					0.23
**m-AAPPQ – Empathy**	−0.03	0.05	−0.04	0.46	(−0.13, 0.06)					−0.6
**AQ-9 Stigma**	0	0.01	0.02	0.89	(−0.02, 0.03)					0.4
Step 3						0.56	0.53	0.04	20.26 (11,176), *p* < 0.001	
Constant	2.09	0.52		<0.001	(1.47, 3.50)					4.06
TPB Attitudes	0.61	0.1	0.57	<0.001	(0.34, 0.84)					6.46
TPB Subjective Norms	0.14	0.06	0.14	0.03	(0.02, 0.31)					2.22
TPB Perceived behavioral control	0.2	0.09	0.19	0.18	(−0.07, 0.32)					2.22
m-AAPPQ – Therapeutic commitment	−0.1	0.12	−0.08	0.69	(−0.29, 0.22)					−0.81
m-AAPPQ – Role security	0.04	0.1	0.04	0.85	(−0.21, 0.23)					0.41
m-AAPPQ – Empathy	−0.04	0.05	−0.05	0.35	(−0.15, 0.05)					−0.87
AQ-9 Stigma	0.01	0.01	0.03	0.77	(−0.02, 0.03)					0.58
**Worked with 10 or more voice-hearers**	−0.29	0.13	−0.12	0.02	(−0.50, 0.10)					−2.17
**Personal experience with voice-hearing**	0.19	0.1	0.10	0.14	(−0.05, 0.35)					1.93
**Formal training on voice-hearing** ^**a**^	−0.61	0.29	−0.30	<0.001	(−1.23, −0.23)					−2.06
**No formal training but considerable experience on voice-hearing** ^**a**^	−0.66	0.29	−0.32	<0.001	(−1.29, −0.26)					−2.25

a The reference category for training on helping voice-hearers the group without formal training nor considerable experience. *R*^2^, proportion of the variance explained; *F*, F-ratio; *t*, *t*-test; CI, confidence intervals. TPB = Theory of Planned Behavior; m-AAPPQ = modified Alcohol and Alcohol Problems Perception Questionnaire; AQ-9 = Attribution Questionnaire-9. 95% bias corrected and accelerated confidence intervals reported in parentheses. Confidence intervals and standard errors are based on 2000 bootstrapped samples. Variables in bold font represent the added variables in each block of the hierarchical regression.

When potential outlier cases (*N* = 19 for adult mental health, *N* = 1 for CAMHS and *N* = 6 for EIP) were excluded, the bootstrapped regression showed similar results for the adult mental health group, whereas in the CAMHS group, perceived behavioral control became a significant predictor of intention with *B*(*SE*) = 0.28 (0.10), β = 0.30, *t* = 2.87, *p* = 0.03, BCa95% [0.02, 0.49] and in the EIP group, the TPB subjective norms no longer significantly predicted intention with *B*(*SE*) = 0.10 (0.06), β = 0.10, *t* = 1.62, *p* = 0.05, BCa95% [0, 0.36].

For the primary care clinicians, irrespective of target patient age, the significant predictors of intention to assess distressing voice-hearing were TPB attitudes, *f* ^2^ = 0.03 for adult and *f* ^2^ = 0.06 for young patients, subjective norms, *f* ^2^ = 0.03 for adult and *f* ^2^ = 0.01 for young patients, and perceived behavioral control, *f* ^2^ = 0.02 for adult and *f* ^2^ = 0.03 for young patients. The final model explained 71% of the variance in intention to assess voice-hearing in adult patients, with TPB variables accounting for 95.8, and 77% of the variance in this intention in 12–18-year-olds, with TPB variables accounting for 98.7% (Table 6, 7).

**Table 6:** Linear model of predictors of TPB intention to assess distressing voice-hearing after disclosure of the experience in Primary care clinicians who completed the adult patient version of the survey (*N* = 158).

Variable	*b*	*SE B*	*β*	*p*	*95%CI for b*	*R*^2^	*Adjusted* ^a^	*ΔR*^2^	*F(df)*	*t*
							*R*^2^			
Step 1						0.68	0.68	0.68	100.42 (3,140), *p* < 0.001	
Constant	0.34	0.29		0.44	(−0.25, 0.92)					1.14
TPB Attitudes	0.48	0.12	0.38	0.001	(0.24, 0.71)					3.95
TPB Subjective Norms	0.23	0.08	0.22	0.003	(0.07, 0.40)					2.81
TPB Perceived behavioral control	0.34	0.12	0.29	0.03	(0.11, 0.58)					2.93
Step 2						0.69	0.68	0.01	44.15 (7, 136), *p* < 0.001	
Constant	−0.27	0.6		0.55	(−1.68, 0.96)					−0.44
TPB Attitudes	0.48	0.13	0.38	0.001	(0.16, 0.75)					3.81
TPB Subjective Norms	0.24	0.09	0.23	0.002	(0.13, 0.52)					2.87
TPB Perceived behavioral control	0.41	0.12	0.34	0.01	(0.09, 0.60)					3.3
**m-AAPPQ – Therapeutic commitment**	0.26	0.14	0.13	0.13	(−0.05, 0.59)					1.81
**m-AAPPQ – Role security**	−0.2	0.11	−0.15	0.12	(−0.43, 0.01)					−1.77
**m-AAPPQ – Empathy**	−0.07	0.07	−0.05	0.46	(−0.23, 0.14)					−0.92
**AQ-9 Stigma**	0.01	0.01	0.03	0.56	(−0.01, 0.03)					0.58
Step 3						0.71	0.68	0.01	29.02 (11,132), *p* < 0.001	
Constant	−0.18	0.63		0.64	(−1.69, 1.11)					−0.29
TPB Attitudes	0.48	0.13	0.38	0.002	(0.16, 0.74)					3.71
TPB Subjective Norms	0.22	0.09	0.21	0.005	(0.10, 0.52)					2.56
TPB Perceived behavioral control	0.42	0.12	0.36	0.009	(0.10, 0.62)					3.45
m-AAPPQ – Therapeutic commitment	0.24	0.15	0.12	0.15	(−0.07, 0.56)					1.62
m-AAPPQ – Role security	−0.22	0.11	−0.16	0.12	(−0.46, 0.01)					−1.91
m-AAPPQ – Empathy	−0.03	0.08	−0.02	0.72	(−0.21, 0.17)					−0.41
AQ-9 Stigma	0.01	0.01	0.03	0.59	(−0.02, 0.03)					0.5
**Worked with 10 or more voice-hearers**	0.16	0.17	0.05	0.20	(−0.11, 0.49)					0.94
**Personal experience with voice-hearing**	−0.39	0.2	−0.1	0.12	(−0.64, 0.09)					−1.99
**Formal training on voice-hearing** ^**a**^	−0.1	0.22	−0.03	0.44	(−0.50, 0.22)					−0.48
**No formal training but considerable experience on voice-hearing** ^**a**^	0.06	0.19	0.02	0.84	(−0.32, 0.47)					0.30

a The reference category for training on helping voice-hearers the group without formal training nor considerable experience. *R*^2^, proportion of the variance explained; *F*, F-ratio; *t,* t-test; CI, confidence intervals. TPB, Theory of Planned Behavior; m-AAPPQ = modified Alcohol and Alcohol Problems Perception Questionnaire; AQ-9, Attribution Questionnaire-9. 95% bias corrected and accelerated confidence intervals reported in parentheses. Confidence intervals and standard errors are based on *N* = 2000 bootstrapped samples. Variables in bold font represent the added variables in each block of the hierarchical regression.

**Table 7:** Linear model of predictors of TPB intention to assess distressing voice-hearing after disclosure of the experience in Primary care clinicians who completed the young people version of the survey (*N* = 160).

Variable	*b*	*SE B*	*β*	*p*	*95%CI for b*	*R*^2^	*Adjusted* ^a^	*ΔR*^2^	*F(df)*	*t*
							*R*^2^			
Step 1						0.77	0.76	0.77	144.44 (3,133), *p* < 0.001	
Constant	−1.04	0.31		0.008	(−1.73, −0.03)					−3.42
TPB Attitudes	0.7	0.11	0.47	<0.001	(0.48, 0.88)					6.44
TPB Subjective Norms	0.18	0.08	0.14	0.047	(0.002, 0.27)					2.16
TPB Perceived behavioral control	0.48	0.11	0.33	<0.001	(0.27, 0.74)					4.47
Step 2						0.77	0.76	0.01	62.68 (7, 129), *p* < 0.001	
Constant	−0.69	0.53		0.24	(−1.89, 0.43)					−1.31
TPB Attitudes	0.69	0.12	0.47	<0.001	(0.47, 0.88)					5.97
TPB Subjective Norms	0.2	0.09	0.15	0.03	(0.02, 0.39)					2.35
TPB Perceived behavioral control	0.54	0.12	0.38	<0.001	(0.30, 0.84)					4.5
**m-AAPPQ – Therapeutic commitment**	0.04	0.17	0.02	0.77	(−0.30, 0.41)					0.24
**m-AAPPQ – Role security**	−0.1	0.12	−0.07	0.42	(−0.36, 0.14)					−0.89
**m-AAPPQ-Empathy**	−0.05	0.08	−0.04	0.42	(−0.20, 0.08)					−0.7
**AQ-9 Stigma**	−0.02	0.01	−0.07	0.16	(−0.04, 0.01)					−1.49
Step 3						0.77	0.75	0.0004	38.74 (11, 125), *p* < 0.001	
Constant	−0.65	0.55		0.27	(−1.91, 0.50)					−1.19
TPB Attitudes	0.69	0.12	0.47	<0.001	(0.48, 0.89)					5.86
TPB Subjective Norms	0.2	0.09	0.15	0.04	(0.01, 0.40)					2.26
TPB Perceived behavioral control	0.53	0.13	0.37	<0.001	(0.30, 0.85)					4.24
m-AAPPQ – Therapeutic commitment	0.03	0.18	0.02	0.83	(−0.32, 0.40)					0.19
m-AAPPQ – Role security	−0.09	0.13	−0.07	0.57	(−0.38, 0.23)					−0.73
m-AAPPQ-Empathy	−0.05	0.08	−0.04	0.45	(−0.20, 0.09)					−0.69
AQ-9 Stigma	−0.02	0.01	−0.07	0.17	(−0.04, 0.01)					−1.46
**Worked with 10 or more voice-hearers**	0.04	0.17	0.01	0.64	(−0.22, 0.36)					0.24
**Personal experience with voice-hearing**	−0.01	0.18	0	0.95	(−0.34, 0.28)					−0.03
**Formal training on voice-hearing** ^**a**^	0.01	0.22	0	0.76	(−0.43, 0.28)					0.04
**No formal training but considerable experience on voice-hearing** ^**a**^	−0.06	0.2	−0.02	0.64	(−0.49, 0.25)					−0.3

a The reference category for training on helping voice-hearers the group without formal training nor considerable experience. *R*^2^, proportion of the variance explained; F, F-ratio; *t*, *t*-test; CI, confidence intervals. TPB = Theory of Planned Behavior; m-AAPPQ = modified Alcohol and Alcohol Problems Perception Questionnaire; AQ-9 = Attribution Questionnaire-9. 95% bias corrected and accelerated confidence intervals reported in parentheses. Confidence intervals and standard errors are based on *N* = 2000 bootstrapped samples. Variables in bold font represent the added variables in each block of the hierarchical regression.

When potential outlier cases (*N* = 2 and *N* = 1 for the primary care clinicians adult patients and young people target patients, respectively) were excluded, TPB perceived behavioral control was no longer a significant predictor of intention to assess voice-hearing in adult patients, although it remained at trend level; *B*(*SE*) = 0.37 (0.12), *β* =0.32, *t* = 2.97, *p* = 0.05, BCa95% [0.01, 0.56]. Stigma (AQ-9) became marginally a significant predictor of intention to assess voice-hearing in young people, with stigma inversely linked to intention, *B*(*SE*) = − 0.03 (0.01), β = −0.10. *t* = −2.15, *p* = 0.04, BCa95% [−0.05, −0.001].

When including all service groups (*N* = 1751), type of service, *f*-s^2^ < 0.02, more positive attitudes, *f* ^2^ = 0.03, greater subjective norms, *f* ^2^ = 0.03, greater perceived behavioral control, *f* ^2^ = 0.02, and greater empathy, *f* ^2^ = 0.002, significantly predicted intention to assess distressing voice-hearing (Table 8). Compared to adult mental health services, EIP and CAMHS clinicians had greater intention to assess voice-hearing, *f* ^2^ < 0.02. The final model provided significant fit to the data overall, with predictors explaining 60% of the intention to assess voice-hearing. Most of the variance in the model seemed to be explained by the TPB measures (52%).

**Table 8:** Linear model of predictors of TPB intention to assess distressing voice-hearing after disclosure of the experience for all participants (N = 1751).

Variable	*b*	*SE B*	*β*	*p*	*95%CI for b*	*R*^2^	*Adjusted*^a^ *R*^2^	Δ*R*^2^	*F(df)*	*t*
Step 1						0.07	0.07	0.07	36.58 (3,1,387), *p* < 0.001	
Constant	5.75	0.05		<0.001	(5.69, 5.87)					127.38
EIP^a^	0.55	0.1	0.15	<0.001	(0.41, 0.73)					5.59
CAMHS^a^	0.27	0.11	0.07	0.02	(0.04, 0.42)					2.5
Primary care^a^	−0.62	0.09	−0.19	<0.001	(−0.83, −0.43)					−6.87
Step 2						0.60	0.60	0.52	341.81 (6,1,384), *p* < 0.001	
Constant	0.34	0.13		0.029	(0.06, 0.73)					2.52
EIP^a^	0.17	0.07	0.05	0.002	(0.07, 0.31)					2.58
CAMHS^a^	0.31	0.07	0.08	<0.001	(0.20, 0.47)					4.38
Primary care^a^	0.15	0.06	0.05	0.006	(0.03, 0.27)					2.45
**TPB Attitudes**	0.44	0.04	0.35	<0.001	(0.34, 0.53)					11.07
**TPB Subjective Norms**	0.27	0.03	0.23	<0.001	(0.21, 0.34)					9.57
**TPB Perceived behavioral control**	0.31	0.04	0.27	<0.001	(0.20, 0.40)					8.62
Step 3						0.60	0.60	0.004	208. 27 (10, 1,380), *p* < 0.001	
Constant	0.29	0.2		0.16	(−0.11, 0.78)					1.44
EIP^a^	0.19	0.07	0.05	0.002	(0.09, 0.32)					2.85
CAMHS^a^	0.31	0.07	0.08	0.001	(0.20, 0.47)					4.38
Primary care^a^	0.12	0.07	0.04	0.044	(0.004, 0.27)					1.76
TPB Attitudes	0.46	0.04	0.36	<0.001	(0.35, 0.55)					10.91
TPB Subjective Norms	0.26	0.03	0.23	<0.001	(0.21, 0.34)					9.46
TPB Perceived behavioral control	0.35	0.04	0.30	<0.001	(0.23, 0.43)					9.11
**m-AAPPQ – Therapeutic commitment**	0.07	0.05	0.05	0.19	(−0.04, 0.18)					1.41
**m-AAPPQ – Role security**	−0.08	0.04	−0.07	0.10	(−0.15, 0.01)					−2.11
**m-AAPPQ – Empathy**	−0.06	0.02	−0.06	0.007	(−0.11, −0.01)					−2.83
**AQ-9 Stigma**	0	0	0.01	0.55	(−0.01, 0.01)					0.79
Step 4						0.60	0.60	0.0003	148.54 (14,1,376), *p* < 0.001	
Constant	0.28	0.21		0.17	(−0.14, 0.78)					1.36
EIP^a^	0.2	0.07	0.05	0.002	(0.10, 0.33)					2.93
CAMHS^a^	0.31	0.07	0.08	0.001	(0.20, 0.48)					4.4
Primary care^a^	0.13	0.07	0.04	0.054	(−0.001, 0.26)					1.76
TPB Attitudes	0.46	0.04	0.36	<0.001	(0.36, 0.55)					10.88
TPB Subjective Norms	0.26	0.03	0.23	. < 0.001	(0.21, 0.33)					9.35
TPB Perceived behavioral control	0.35	0.04	0.30	<0.001	(0.23, 0.43)					9.06
m-AAPPQ – Therapeutic commitment	0.07	0.05	0.05	0.21	(−0.04, 0.18)					1.42
m-AAPPQ – Role security	−0.08	0.04	−0.07	0.16	(−0.15, 0.02)					−2.01
m-AAPPQ – Empathy	−0.06	0.02	−0.05	0.01	(−0.10, −0.01)					−2.65
AQ-9 Stigma	0	0	0.01	0.56	(−0.01, 0.01)					0.77
**Worked with 10 or more voice-hearers**	0	0.06	0	0.77	(−0.10, 0.15)					−0.08
**Personal experience with voice-hearing**	−0.04	0.05	−0.01	0.54	(−0.14, 0.07)					−0.74
**Formal training on voice-hearing** ^**b**^	0.01	0.09	0	0.52	(−0.22, 0.13)					0.08
**No formal training but considerable experience on voice-hearing** ^**b**^	0.04	0.08	0.01	0.96	(−0.17, 0.17)					0.48

a The reference category for these dummy variables that represent the type of clinicians’ service was Adult Mental Health services.

b The reference category for training on helping voice-hearers was the group without formal training nor considerable experience. *R*^2^, proportion of the variance explained; *F*, F-ratio; *t*, *t*-test; CI, confidence intervals. TPB, Theory of Planned Behavior; m-AAPPQ, modified Alcohol and Alcohol Problems Perception Questionnaire; AQ-9, Attribution Questionnaire-9. 95% bias corrected and accelerated confidence intervals reported in parentheses. Confidence intervals and standard errors are based on 2000 bootstrapped samples. Variables in bold font represent the added variables in each block of the hierarchical regression.

### 3.3. Aim 3: the effect of TPB beliefs-based measures on intention to assess distressing voice-hearing in young people.

Clinicians were split into no or low intention versus moderate or high intention to assess distressing voice-hearing based on their mean TPB intention score, with scores ranging from 1 to 5 indicating no to low intention and 6 to 7 moderate to high intention. Based on the principle of parsimony (Field, 2017), five weighted beliefs, two behavioral, two normative and one control belief for CAMHS, and four behavioral beliefs and one normative belief for EIP clinicians, were retained in the final logistic regression models. The overall model accuracy of predicting clinicians’ intention group based on their belief scores was at 86.4% (78.9% for the null model) for CAMHS and 91.8% (84% for the null model) for EIP clinicians.

For EIP and CAMHS clinicians, the behavioral belief that assessing voice-hearing would help with constructing a detailed formulation of the young person’s presentation significantly increased the likelihood of having a moderate/high intention to assess voice-hearing. In CAMHS, a one-point increase in this belief increased the odds of having high/medium intention to assess group by 32% (Table 9) and in EIP by 84%. Similarly, the normative belief that specialist mental health professionals think that they should assess distressing voice-hearing after disclosure was associated with clinicians having moderate/high intention to assess voices in both service groups. In CAMHS, a one-point increase in this normative belief increased the likelihood of clinicians belonging in the moderate/high group by 20% (*p* = 0.002) (Table 9) and for EIP clinicians by 39% (*p* < 0.001) (Table 10). Specifically, in CAMHS, the control belief that having voice-hearing assessment tools are available in their day-to-day clinical practice was positively associated with higher likelihood of clinicians reporting moderate/high intention to assess voice-hearing in young people (*p* = 0.036) (Table 9). However, no significant associations were found between intention and beliefs about whether assessing voice-hearing would lead to mistakenly labeling the young person with a mental health disorder such as psychosis or whether the clinician believes the young person thinks they should assess their voice-hearing experiences, *p*s > 0.05. Among EIP clinicians, those who believed less intensely that assessing voice-hearing would lead to over-focusing on voices and incomplete exploration of other critical areas of a young person’s presentation were more likely to belong in the moderate/high intention group *(p* = 0.001) (Table 10).

**Table 9:** Summary of binary logistic regression examining the effect of indirect TPB weighted beliefs on TPB intention for CAMHS clinicians.

Variables	Intention group		*b* (*SE*)	Wald statistic	*p*	*OR*^a^ (95% CI)
	No /Low (*N* = 32)	Moderate/High (*N* = 116)				
	*M* (*SD*)	*M* (*SD*)				
Constant			−4.10 (2.25)	3.34		0.04
Assessing voice-hearing would help with constructing a detailed formulation of what is happening for the young person	18.19 (2.56)	19.66 (1.87)	0.27 (0.11)	5.78	0.015	1.32 (1.05, 1.64)
Assessing voice-hearing would lead to mistakenly labeling the young person with a mental health disorder such as psychosis	−8.78 (4.35)	−6.07 (3.68)	0.13 (0.06)	4.06	0.061	1.14 (1.00, 1.29)
The young person thinks I should assess their voice-hearing experiences	0.38 (5.92)	5.85 (6.49)	0.02 (0.06)	0.12	0.678	1.02 (0.91, 1.14)
Specialist mental health practitioners (e.g., psychologists, psychiatrists) think I should assess the young person’s voice-hearing experiences	−2.91 (9.00)	9.26 (6.70)	0.18 (0.04)	16.42	0.002	1.20 (1.10, 1.30)
Voice-hearing assessment tools (e.g., assessment measures, questionnaires) are available to me.	3.88 (6.88)	5.85 (5.36)	0.11 (0.05)	5.14	0.036	1.12 (1.02–1.23)

a The no/low intention group was used as reference. *R*^2^ = 0.35(Cox-Snell), 0.55 (Nagelkerke), Model χ^2^(5) = 63.75, *p* < 0.001. *OR* = odds ratio, *b* = regression coefficient on the independent variable, CI, confidence interval. Significance values are based on *N* = 2000 BCa 95% bootstrapped samples.

**Table 10:** Summary of binary logistic regression examining the effect of indirect TPB weighted beliefs on TPB intention for EIP clinicians.

	Intention group					
	No /Low (*N* = 31)	Moderate/High (*N* = 163)				
Variables	*M* (*SD*)	*M* (*SD*)	*b* (*SE*)	Wald statistic	*p*	*OR*^a^ (95% CI)
Constant			−15.86 (4.82)	10.84	0.001	0
Assessing voice-hearing would help with constructing a detailed formulation of what is happening for the young person	17.32 (3.35)	20.06 (1.58)	0.61 (0.19)	10.13	0.002	1.84 (1.27, 2.69)
Assessing voice-hearing would put engagement with the young person at risk.	11.29 (1.68)	12.6 (1.46)	0.03 (0.07)	0.24	0.709	1.04 (0.91, 1.19)
Assessing voice-hearing would lead to over-focusing on voices and incomplete exploration of other critical areas of a young person’s presentation.	−7.29 (5.46)	−4.26 (3.41)	0.27 (0.10)	7.65	0.001	1.32 (1.08, 1.60)
Assessing voice-hearing would help evaluate the impact of voices on the young person’s functioning	17.32 (2.89)	19.66 (1.57)	0.30 (0.22)	1.81	0.111	1.35 (0.87, 2.09)
Specialist mental health practitioners (e.g., psychologists, psychiatrists) think I should assess the young person’s voice-hearing experiences	−1.42 (9.57)	11.77 (5.19)	0.33 (0.07)	21.19	. < 0.001	1.39 (1.21–1.59)

a The no/low intention group was used as reference. *R*^2^ = 0.43(Cox-Snell), 0.73 (Nagelkerke), Model χ^2^(5) = 107.77, *p* < 0.001. OR, odds ratio, *b* = regression coefficient on the independent variable, CI, confidence interval. Significance values are based on *N* = 2000 BCa 95% bootstrapped samples.

When the analysis was repeated for EIP clinicians without potential outlier cases (*N* = 32), an additional behavioral belief was also found to contribute significantly to the model (the belief that assessing voice-hearing would help evaluate the impact of voices on the young person’s functioning, *OR* = 2.25, 95% CI [0.47–10.86], Wald statistic = 1.02, *p* = 0.004). Re-running the analysis for CAMHS clinicians without potential outlier cases (*N* = 12) resulted in only one predictor belief contributing significantly to the resulting model (normative belief about specialist mental health professionals approving of their assessing voice-hearing; *OR* = 1.28, 95% CI [1.13–1.46], Wald statistic = 14.28, *p* < 0.001, Nagelkerke *R*^2^ = 0.62, Model χ^2^(5) = 62.41, *p* < 0.001). Welch’s t-test results exploring the differences in all 30 weighted beliefs for no/low versus moderate/high intention groups are presented in Supplementary Tables S7, S8.

## 4. Discussion

This study used a TPB framework to explore the factors that influenced the intention of clinicians to assess the distressing voice-hearing experiences of patients. More specifically, interest was focused upon the intention of clinicians working with young people who were experiencing voices. Comparisons were made between clinicians working within EIP, CAMHS and primary care. A broader comparison group was sampled from clinicians working with adults in secondary and primary care services.

EIP clinicians reported more positive attitudes (therapeutic commitment, role security, empathy) toward working with young voice-hearers, higher self-efficacy in voice-hearing practices compared to all other service groups, and similar levels of stigma toward voice-hearing youth compared to other mental health clinicians. In contrast, primary care clinicians reported the opposite results, irrespective of the patient age group. The present study supported the utility of the TPB as a framework for understanding potential influences on clinician’s intention to assess distressing voice-hearing following patient disclosure across service type and patient age groups. Although the addition of background factors, such as job attitudes toward working with voice-hearers, was found to contribute significantly to clinicians’ intention in some service groups, the importance of their contribution was negligible. Specific beliefs relating to the usefulness of assessing voice-hearing and to the social pressure coming from the approval/disapproval of other specialist mental health professionals regarding assessing voice-hearing in 12-18-year-old patients were linked with clinician intention to do so in both CAMHS and EIP clinicians.

Examination of the first aim showed that all service groups reported at least moderate levels of self-efficacy in asking patients if they hear voices and discussing voice-hearing, regardless of the patient age group. The lowest scores in self-efficacy across service groups were about providing useful voice-hearing information. Primary care clinicians had the lowest scores for both adult and young patients in self-efficacy for all voice-hearing practices. Although asking about the presence of voice-hearing or discussing the experience may have become part of mental health clinical practice in recent years (British Psychological Society, 2014), providing information potentially requires clinicians’ active engagement with the experience and access to information that could be helpful for patients. Additionally, mental health clinicians’ moderate self-reported confidence in asking about or discussing voice-hearing does not necessarily mean that they consider such conversations to be appropriate nor that they actually engage in them (Coffey et al., 2004; Coffey and Hewitt, 2008; Harrison et al., 2008; White et al., 2019). However, if clinicians’ confidence translates into asking about the presence of voice-hearing, it could be especially beneficial for the early detection of such experiences in young people who might be skeptical in disclosing them, unless they are asked directly (Mertin and Hartwig, 2004; Kelleher et al., 2014).

All secondary mental health service groups (EIP, CAMHS, adult mental health) reported similar levels of stigma in comparison to each other but these levels were lower compared to primary care clinicians, with a moderate to large effect, irrespective of the patient age group. Previous literature has shown that primary care clinicians tend to report more negative attitudes toward people with psychotic experiences compared to mental health clinicians (Hori et al., 2011; Mittal et al., 2014; Smith et al., 2017). In the present study, almost half of the primary care clinicians had no formal training in supporting voice-hearers and no or limited clinical experience with this patient group. Thus, higher levels of stigmatizing attitudes could possibly be due to having less work experience (Caplan et al., 2016; Al Saif et al., 2019) or due to having fewer positive experiences with this patient group, rather than contact more broadly. Considering that lack of training in supporting this group can be linked to lower levels of confidence in discussing voice-hearing experiences with patients (Kramarz et al., 2020), this could lead to less opportunities for positive contact experiences and building therapeutic rapport that could disconfirm negative stereotypes and reduce stigmatizing views (Couture and Penn, 2003; Jorm et al., 2012). Furthermore, most primary care clinicians in this study (about 81%) did not have personal or familial experience of hearing voices compared to about 68% in mental health professionals, which could be an additional factor for displaying higher levels of stigmatizing attitudes (Sandhu et al., 2019; Oliveira et al., 2020).

Findings on job attitudes toward working with voice-hearers showed differences among service groups, with EIP clinicians reporting the greatest therapeutic commitment, role security and empathy. Concerning working with young voice-hearers, differences between CAMHS and EIP had moderate to large effect sizes for therapeutic commitment and role security and small for empathy. Higher levels of motivation and satisfaction, feeling more adequate in their role, feeling legitimate when engaging in their clinical tasks with this patient group and relating to a greater extent with patients’ experiences, could be intuitively expected for EIP clinicians as they have more training and/or experience working with patients with voice-hearing and other psychotic experiences. All mental health clinicians had higher role security, therapeutic commitment and empathy compared to primary care clinicians, with a moderate to large effect, except for CAMHS who did not differ in empathy from primary care clinicians. The lower positive attitudes in primary care clinicians could be partly attributed to the lack of positive reinforcement when consulting with patients with mental health difficulties that could leave them with low levels of satisfaction in the care they provide (Zolnierek and Clingerman, 2012) and create doubts about their professional credibility (Harrison and Zohhadi, 2005; Brunero et al., 2018). Differences in self-reported empathy between mental health and primary care clinicians could be explained by the increased social contact of the former group with voice-hearers, which could have increased feelings of empathy and allowed for personal connections (Pettigrew and Tropp, 2006; Agrawal et al., 2016; Maranzan, 2016).

Exploring the predictors of clinicians’ intention to assess distressing voice-hearing following disclosure by patients indicated that TPB employed a well-fit model. The three TPB predictors (attitudes, subjective norms, perceived behavioral control) accounted for more than half of the variance in intention that was higher than the 39% of variance explained typically by TPB (Armitage and Conner, 2001). Mean scores indicated that, overall, clinicians reported high intention to assess voices. This finding is comparable to other studies who found mental health clinicians’ intention to discuss the meaning and content of voices moderately high (MacLeod, 2011) and that the majority attended to the content of hallucinations, despite ambivalence in attitudes toward the value of doing so (Aschebrock et al., 2003). Overall, more positive attitudes, more approving subjective norms and greater perceived behavioral control significantly predicted intention to assess distressing voice-hearing. For adult mental health clinicians and primary care clinicians, all three TPB measures were significant predictors of intention to assess voices. By comparison, in relation to young patients, attitudes and subjective norms in EIP and only subjective norms in CAMHS seemed to significantly explain part of the variance in intention.

Focusing on young people 12–18 years of age, this study found that specific beliefs might be linked to CAMHS and EIP clinicians’ intention to assess voice-hearing. These beliefs concerned: 1) that assessing voices would help with constructing a detailed formulation of the young person’s presentation; and 2) that other specialist mental health professionals (e.g., psychiatrists, psychologists) would approve and think clinicians should assess distressing voice-hearing after disclosure of the experience. Specifically, in CAMHS, having voice-hearing assessment tools (e.g., questionnaires) available was positively related with moderate to high intention to assess voice-hearing in young people. In contrast to EIP, where tools might be more easily accessible in routine clinical practice, CAMHS might not have readily available tools that would support exploration of voice-hearing and related experiences.

Previous research has demonstrated that subjective norms are strong predictors of clinicians intention (Perkins et al., 2007; Kelly et al., 2012), highlighting the increased importance that managers and colleagues can play in influencing clinician’s behaviors. Specifically in mental health studies employing TPB to explain clinicians’ intention of using evidence-based practices (e.g., cognitive behavioral therapy for psychosis), social norms and individual attitudes have been strong predictors of intention, with social norms determining whether evidence-based practice will be delivered (Burgess et al., 2017; Lecomte et al., 2018). Research on influences of psychotherapists’ current clinical practice has also emphasized the importance of other clinicians or role models and informal discussions with colleagues as key determinants of their current practice and treatment decisions (Cook et al., 2009). While in this study clinicians’ average ratings on subjective norms seemed to be slightly to moderately approving, a discouraging service culture toward discussing distressing voices could be due to several reasons including lack of confidence (e.g., Coffey and Hewitt, 2008) and practical issues (e.g., lack of staff) that might lead to prioritizing task completion rather than engaging with patients (McMullan et al., 2018; White et al., 2019; McCluskey and Vries, 2020). Specific to young people, having a working culture that deters clinicians from in-depth discussions on voice-hearing might have to do with the experience *per se*; voice-hearing in young people may not be commonly linked with severe mental illness and could potentially be considered as part of normal development (Maijer et al., 2019) thus dismissed.

Perceived behavioral control did not significantly predict intention to assess voices in young people, although it seemed to predict clinicians’ intention overall. According to a meta-analysis (Notani, 1998), perceived behavioral control is often a poor predictor of intention when the target behavior is relatively unfamiliar to the participants, as one might need an adequate level of actual experiences in order to truly appreciate the barriers involved in achieving the target behavior. Since assessment of distressing voices in young people might be an unfamiliar behavior for clinicians, their perceptions of control may be based on unrealistic assumptions.

### 4.1. Strengths and limitations

This is the first study to employ TPB to understand the influences on clinician’s intention to discuss distressing voice-hearing in young people and one of the few studies to explore staff views on that subject. Previous studies have focused mostly on mental health acute wards and smaller samples, usually of nurses (Coffey and Hewitt, 2008; McMullan et al., 2018). The study had a relatively large sample size when compared to other studies and had sufficient statistical power. Moreover, it included a range of clinicians from both primary care and secondary mental health services, from multiple regions, generating a representative UK sample of staff from healthcare.

The study did, however, have several limitations. Although asking CAMHS clinicians to answer questions about patients aged 12–18 would refer to a commonly treated age group, it is possible that EIP clinician exposure to this age group is very limited. According to the 2019–2010 National Clinical Audit of Psychosis (Royal College of Psychiatrists, 2020), patients under 18 years of age only constituted 1.8% of the caseload for UK EIP services. This might have influenced clinicians’ responses, and the reliability and validity of findings given the sample size for this service group. Rigidity of professional boundaries could be another factor to consider when interpreting clinicians’ responses. Some clinicians might not have viewed assessing voice-hearing as be part of their professional role (e.g., health care assistants, students). This is supported by our finding that 15.7% of the overall sample reported that they do not conduct patient mental health assessments as part of their current role. However, the definition of “assessment” given in the TPB questionnaire reflected an in-depth detailed conversation about distressing voice-hearing experience, and did not refer to a formal psychiatric assessment, which most clinical staff should be able to engage with as part of their role. Finally, the modified AAPPQ (McLeod et al., 2002) and the AQ-9 (Corrigan et al., 2014), were adapted to capture clinicians’ attitudes toward working with patients who hear distressing voices, and the TPB and voice-hearing practice self-efficacy questionnaires were developed for this study. Although the internal consistency of all measures was examined and deemed acceptable, further psychometric testing is needed to ensure the reliability of those measures in the context of clinicians working with voice-hearers.

### 4.2. Future directions

Future studies should aim to examine voice-hearing practices, rather than focusing on behavioral intention. Despite evidence that intention is a moderate predictor of clinician self-reported behavior (Godin et al., 2000; Eccles et al., 2006), other factors might mediate the relationship between behavioral intention and implementation. Perkins et al. (2007) indicated that even in cases where clinician intention is high to perform a goal-directed behavior, there might be other obstacles encountered en route to behavioral performance (e.g., habits and automatic processes, behavioral skills and cues). Additionally, studies have found different TPB components to predict behaviors depending on clinicians’ professional group membership and their specific norms (Perkins et al., 2007; Hrisos et al., 2009; Kortteisto et al., 2010). Thus, research on guiding the implementation of changes regarding clinicians’ behavior toward patients with distressing voices might be worth focusing on specific service groups. Considering the important role of subjective norms in predicting clinicians’ behavior, research on interventions aiming to increase intention to discuss distressing voices could improve understanding on the most effective forms of social influence within health services.

### 4.3. Implications

Considering the modest clinician confidence in providing useful information to patients with distressing voice-hearing, offering more knowledge on this experience to clinicians might increase their confidence to facilitate discussions. As a lack of material support and resources has been identified as one of the key barriers to the translation of knowledge into healthcare practice (Cochrane et al., 2007), having access to material and resources (e.g., psychoeducation leaflets, questionnaires) could support clinicians to engage in conversations about voice-hearing. Regarding young people, any information should be developmentally appropriate, and clinicians’ responsiveness should be tailored to their developmental stage to enhance engagement with this patient group (Jones et al., 2017).

The promotion of in-depth conversations between clinicians and patients about distressing voice-hearing may also benefit from changes in the work environment. Rather than intervening to alter clinicians’ job attitudes toward working with voice-hearers or specific attitudes on assessment, training professionals who set the example or are highly appreciated within a service could be beneficial. This could refer to specialist or senior mental health professionals who facilitate supervision and are responsible for team training activities. They could stimulate conversations about voice-hearing within clinical teams and promote a view that talking about voices is approved by peers and could be beneficial to patients (Coffey and Hewitt, 2008). Clinician training about continuing a discussion about voice-hearing could focus upon using an open and non-judgmental approach (Romme et al., 2009; Griffiths et al., 2019), and employing curiosity about the young person’s beliefs about their experience considering their cultural or personal frames of reference (Coughlan et al., 2022). The responses of other people to the young person in relation to voice-hearing and the young person’s perceptions of these responses should also be explored as they could influence the distress and the nature of the voices (Parry et al., 2020). Subsequent training of selected clinicians within a service could focus upon the delivery of brief and targeted therapy for voices, such as Coping Strategy Enhancement (Hayward et al., 2018) which has been evaluated as an acceptable and helpful way for young people to initiate a therapeutic conversation about their distressing voices (Hayward et al., 2022).

Acknowledging that some clinicians might find it difficult to explore the nature of young people’s voice-hearing experiences (Byrne et al., 2020), the use of structured tools in young people’s services might reduce uncertainty among clinicians and facilitate conversations (Bogen-Johnston et al., 2020). Examples of assessment measures that could stimulate and guide conversations about voice hearing experiences in young people are the Auditory Vocal Hallucination Rating Scale (Jenner and van de Willige, 2002; Bartels-Velthuis et al., 2012), Psychotic Symptom Rating Scales for Auditory Hallucinations (Haddock et al., 1999) and Hamilton Program for Schizophrenic Voices Questionnaire (Van Lieshout and Goldberg, 2007) which have all been used in youth samples (Bartels-Velthuis et al., 2016; Hayward et al., 2022; Rammou et al., 2022).

### 4.4. Conclusion

EIP clinicians had the lowest stigma, most positive job attitudes and highest self-efficacy in voice-hearing practices with young people, while the responses of primary care clinicians demonstrated the opposite. Clinicians’ intention to assess distressing voices in both young and adult patients after disclosure was moderately high, with the TPB variables of attitudes, subjective norms and perceived behavioral control explaining a large part of its variance. Intention toward the facilitation of in-depth discussions about voice hearing in young people were influenced by the practices of specialist mental health colleagues and by beliefs about the contribution that could be made to client formulations. Promoting a work culture that encourages discussions about voice-hearing between colleagues and with patients, as well as introducing supportive material about voices (e.g., questionnaires, psychoeducation leaflets), might have a positive impact upon encouraging discussion about voices, especially in CAMHS.

## Data availability statement

The datasets presented in this article are not readily available because of ethical reasons. Requests to access the datasets should be directed to the corresponding author, ar353@sussex.ac.uk.

## Ethics statement

The study was sponsored by the University of Sussex, UK and received ethical approval from the Health Research Authority (Reference: 048 HAY/ IRAS ID: 257355). The patients/participants provided their written informed consent to participate in this study.

## Author contributions

AR, CB, MH, and DF made substantial contributions to the conception and design of the study. AR was responsible for the data collection and organization. Statistical analysis was led by AR and CB and interpretation was led by AR. The manuscript was drafted by AR, with significant contributions from CB, MH, and DF. All authors contributed to the article and approved the submitted version.

## Funding

This work was supported by the Economic and Social Research Council (PhD studentship award number ES/J500173/1).

## Conflict of interest

The authors declare that the research was conducted in the absence of any commercial or financial relationships that could be construed as a potential conflict of interest.

## Publisher’s note

All claims expressed in this article are solely those of the authors and do not necessarily represent those of their affiliated organizations, or those of the publisher, the editors and the reviewers. Any product that may be evaluated in this article, or claim that may be made by its manufacturer, is not guaranteed or endorsed by the publisher.

## Abbreviations

TPB: Theory of Planned Behavior
CAMHS: Child and Adolescent Mental Health Services
EIP: Early Intervention in Psychosis Services
NHS: National Health Service
