# Supplementary material for: “Attitudes to voices”: a survey exploring the factors influencing clinicians’ intention to assess distressing voices and attitudes towards working with young people who hear voices
Source: Front Psychol. 2023 May 23;14:1167869. doi: 10.3389/fpsyg.2023.1167869 (PMC10242135; doi:10.3389/fpsyg.2023.1167869)
Supplement: Supplementary file 1 [file Table_1.DOCX]

Supplementary Material

1. **Supplementary Method information**

**Method S1.** *Development of the indirect TPB belief measures*

To develop the indirect TPB measures, an elicitation phase took place to identify commonly held beliefs about assessing distressing voice-hearing among CAMHS and EIP clinicians. During this phase *N* = 25 CAMHS and EIP clinicians completed a short self-report questionnaire with open-ended questions aiming: 1) to identify the content of beliefs about the behavior under examination (advantages and disadvantages of performing the behavior); 2) to identify groups and categories of individuals (‘reference groups’) who are likely to apply social pressure (approve or disapprove) with respect to the behavior and; 3) to identify the content of control beliefs (enablers and barriers) about the behavior. These indirect measures are presumed to determine the more global reactions of the direct measures and are central to TPB as they provide the cognitive and affective foundations for attitudes, subjective norms, and PBC (Ajzen, 1991). Through content analysis of their responses, the behavioral beliefs, the groups that apply social pressure and the control beliefs most often listed were selected and converted into a set of statements. Two independent researchers proceeded with content analysis of the responses into themes and labelled the themes extracted. Both researchers carried out these steps independently to increase the validity of the analysis. The extracted themes were listed from the most frequently expressed to the least frequently expressed and 75% of all beliefs/statements were included, considering they provide adequate coverage of the pool of beliefs of the target population (Francis et al., 2004). For each belief items, an outcome evaluation item was created.

The questionnaire went through face validity testing with 5 CAMHS and EIP clinicians and then it was piloted with 14 CAMHS and EIP clinicians. Indirect items that did not have any variance in their response score were removed. Direct items that did not have any variance or had significant skewness (*SE* > 1.96) were removed. Cronbach’s alpha for all direct items was calculated and items were removed if their removal improved scale reliability.

*Scoring of indirect TPB measures*

For the indirect measure of attitudes, each of the 12 behavioral beliefs which represented a particular outcome of assessing voice-hearing (rated on a 7-point Likert scale, extremely unlikely to extremely likely) was multiplied with evaluations of outcomes (rated on -3 to +3 bipolar scale from extremely bad to extremely good). Therefore, each pair of items produced a single datum from −21 to +21. For some of the behavioral beliefs (e.g., “Doing something positive for the patients is…”), outcome evaluation items were omitted as they exhibited little variance in the pilot study (see Appendix F, Supplementary Table 3 for example items). These items were replaced with a constant, based on the most frequently selected response option by the pilot participants (Francis et al., 2004). For the indirect measure of subjective norms, 5 normative beliefs were multiplied with items indicating clinicians’ motivation to comply. For the indirect measure of perceived behavioral control, 13 control beliefs were multiplied with perceived power of beliefs these beliefs. Then, total scores of the weighted beliefs were calculated to represent a composite score for attitude, subjective norms, and perceived behavioral control.

Validity of indirect measures

A series of Pearson’s bivariate correlations were calculated between direct and indirect measures of the same TPB constructs, to confirm the validity of the indirect measures in CAMHS (N = 160) and EIP (N = 207). All indirect TPB measures correlated significantly with their respective direct measures (ps <.001), with Pearson’s r correlation coefficients ranging from r = .45 to r = .62.

**Table S1.** *The indirect TPB belief items included in the TPB questionnaire section of the survey addressed to CAMHS and EIP clinicians.*

| Type of Belief | Beliefs |
| --- | --- |
| Behavioral Beliefs | **When a young person discloses hearing distressing voices to me assessing their voice-hearing experiences…** |
|  | would help with constructing a detailed formulation of what is happening for the young person. |
|  | would put engagement with the young person at risk. |
|  | would help identify the right support or treatment if needed. |
|  | would help promote good engagement between me and the young person |
|  | would lead to over-focusing on voices and incomplete exploration of other critical areas of a young person’s presentation. |
|  | would aid diagnosing. |
|  | would be reassuring and validating for the young person |
|  | would make the young person feel distressed (e.g., anxious, fearful). |
|  | would help with assessing the risk to self/others that is related to voices. |
|  | would lead to mistakenly labelling the young person with a mental health disorder such as psychosis |
|  | would help evaluate the impact of voices on the young person’s functioning |
|  | would help evaluate the impact of voices on the young person’s emotions (e.g., distress). |
| Normative beliefs | **When a young person discloses hearing distressing voices to me...** |
|  | the young person thinks I should assess their voice-hearing experiences |
|  | Specialist mental health practitioners (e.g., psychologists, psychiatrists) think I should assess the young person’s voice-hearing experiences |
|  | individuals in the young person’s social system (e.g., family, friends) think I should assess the young person’s voice-hearing experiences. |
|  | other colleagues in my clinical team would assess the young person’s voice-hearing experiences. |
|  | clinicians in the same profession as me would assess the young person's voice-hearing experiences |
| Control beliefs | **In my routine clinical practice…** |
|  | I have limited time to assess young people’s experiences. |
|  | I can find a suitable space to assess young people’s experiences. |
|  | young people who hear distressing voices are very unwell to engage in an assessment of their voice-hearing experiences. |
|  | I have good engagement with young people who hear distressing voices. |
|  | young people present with practical issues (e.g., lack of permanent address, current life stressors) that seem more immediate than their voice-hearing experiences. |
|  | young people who disclose hearing distressing voices to me are unwilling to discuss their voice-hearing experiences with me. |
|  | young people who hear distressing voices present with high-risk issues. |
|  | voice-hearing assessment tools (e.g., assessment measures, questionnaires) are available to me. |
|  | people in the social system of young people (e.g., carers, friends) are accepting of the young person’s voice-hearing experiences. |
|  | I have the opportunity to consult my team on how to proceed with my cases. |
|  | young people who hear distressing voices do not have the ability to answer assessment questions relating to their voice-hearing experiences. |
|  | I have access to adequate collateral information (e.g., family history, young person’s culture, values) about the young people I am working with. |
|  | I have had training or knowledge in assessing voice-hearing in young people |

**Table S2**. *Example questionnaire items for the TPB indirect measures*

| Belief strength | Number of items | Example item | Impact of beliefs | Number of items | Example item |
| --- | --- | --- | --- | --- | --- |
| Attitudes | 12 | When a young person discloses hearing distressing voices to me...  assessing their voice-hearing experiences would put engagement with the young person at risk.  Response scale: Extremely unlikely- Extremely likely(7-point Likert scale) | Outcome evaluation | 5 | Putting engagement with a young person at risk is...  Response scale: Extremely bad- Extremely good (bipolar scale -3 to +3) |
| Perceived norms | 5 | When a young person discloses hearing distressing voices to me, specialist mental health practitioners (e.g., psychologists, psychiatrists) think I should assess the young person’s voice-hearing experiences.  Response scale: Extremely unlikely- Extremely likely (7-point Likert scale) | Motivation to comply | 5 | In general, when it comes to my clinical practice, I want to do what specialist mental health practitioners (e.g., psychologists, psychiatrists) think I should do  Response scale: Very strongly disagree to Very strongly agree (bipolar scale -3 to +3) |
| Perceived behavioral control | 13 | In my routine clinical practice, I have limited time to assess young people’s experiences.  Response scale: Extremely unlikely- Extremely likely (7-point Likert scale) | Perceived power of beliefs | 13 | When a young person discloses hearing distressing voices to me, having limited time would make it difficult for me to assess their voice-hearing experiences. (reverse-scored item)  Response scale: Very strongly disagree (+3) to Very strongly agree (-3) |

1. **Supplementary Analysis Information**

**2.1 Analysis S1.** *Missing data analysis*

In the whole sample (*N* = 1751), regarding the stigma AQ-9 scale, all three modified AAPPQ scales (therapeutic commitment, role security, empathy), TPB attitudes, subjective norms, perceived behavioral control and intention scales, non-completers were younger, had less years of experience in their current professional role compared to completers (*p*s < .005). The type of service had a significant relationship with the completion of stigma AQ-9 scale, all three modified AAPPQ scales, TPB attitudes, perceived behavioral control and intention (*p*s < .005). For the stigma AQ-9 scale, the three modified AAPPQ scales and TPB intention scale, there were less completers in CAMHS compared to the other service groups and more completers in primary care clinicians who completed the adult patient version compared the other service groups. For the TPB attitudes and perceived behavioral control scales, there were more completers in primary care clinicians who completed the adult patient version of the survey (*p*s < .005). White British clinicians and clinicians who had experience working with 10 or more voice-hearers (compared to have worked with none to 9 voice-hearers) were more likely to have completed the voice-hearing practice self-efficacy scale on providing useful information (*p*s < .005).

Within the adult mental health service group, there were more White British clinicians who completed the TPB subjective norm, TPB perceived behavioral control scales and the voice-hearing practice self-efficacy scale on providing useful information to voice-hearers. Healthcare assistants were more likely to be non-completers of the TPB subjective norms scale compared to all other professions and clinicians who had experience working with 10 or more voice-hearers were more likely to have completed the voice-hearing practice self- efficacy scale on providing useful information compared to clinicians who had worked with none to 9 voice-hearers).

Within the EIP group, non-completers of the stigma AQ-9, modified AAPPQ therapeutic commitment, role security, empathy and TPB intention scales had less years of experience working in mental health services (*p*s < .005). Non-completers of the modified AAPPQ therapeutic commitment, role security, empathy and TPB Intention scales were more likely to be younger compared to completers. Non-completers of the three self-efficacy scales (to ask as patient if they hear voices, to discuss voice-hearing and to provide useful information) had less years of experience in their current role compared to completers (*p*s < .001).

Within the CAMHS group, clinicians with experience working with 10 or more voice-hearers compared to those who had worked with none to 9 voice-hearers were more likely to have completed the TPB attitudes and subjective norm scales. Nationality seemed to have a significant relationship with completing the three voice-hearing practice self-efficacy scales, with more British clinicians having completed the scales compared to those reporting a non-British nationality (*p*s < .005).

Evaluation of missing values within the primary care clinician data revealed that in the group that completed the survey about adult patients, non-completers of the three voice-hearing practice self-efficacy scales had significantly less years of experience working in mental health services compared to completers. Additionally, non-completers of the question on past training in supporting voice-hearers had less experience in their current role compared to completers (*p* < .005).

Regarding the primary care clinicians who completed the young patient version of the survey, non-completers of the question on past training in supporting voice-hearers had less experience working in mental health compared to completers (*p* < .005).

**2.2 Tables**

**Table S3.** *Missing cases and percentages in the main study variables.*

|  | All participants (*N* = 1751) | | Adult Mental health (*N* = 966) | | EIP (*N* = 253) | | CAMHS (*N* = 214) | | Primary Care (Adult version) (*N* = 158) | | Primary Care (Young people version) (*N* = 160) | |
| --- | --- | --- | --- | --- | --- | --- | --- | --- | --- | --- | --- | --- |
|  | *N* | % | *N* | % | *N* | % | *N* | % | *N* | % | *N* | % |
| Stigma AQ9 Total | 354 | 20.2% | 180 | 18.6% | 64 | 25.3% | 73 | 34.1% | 14 | 8.9% | 23 | 14.4% |
| m-AAPPQ Therapeutic Commitment | 345 | 19.7% | 177 | 18.3% | 63 | 24.9% | 70 | 32.7% | 13 | 8.2% | 22 | 13.8% |
| m-AAPPQ Empathy | 341 | 19.5% | 172 | 17.8% | 64 | 25.3% | 70 | 32.7% | 13 | 8.2% | 22 | 13.8% |
| m-APPQ Role Security | 333 | 19.0% | 166 | 17.2% | 63 | 24.9% | 70 | 32.7% | 12 | 7.6% | 22 | 13.8% |
| TPB intention | 301 | 17.2% | 148 | 15.3% | 59 | 23.3% | 66 | 30.8% | 9 | 5.7% | 19 | 11.9% |
| TPB perceived behavioral control | 249 | 14.2% | 143 | 14.8% | 41 | 16.2% | 40 | 18.7% | 8 | 5.1% | 17 | 10.6% |
| TPB attitudes | 245 | 14.0% | 141 | 14.6% | 41 | 16.2% | 38 | 17.8% | 8 | 5.1% | 17 | 10.6% |
| TPB subjective norms | 196 | 11.2% | 113 | 11.7% | 32 | 12.6% | 29 | 13.6% | 8 | 5.1% | 14 | 8.8% |
| Self-efficacy to provide useful information | 102 | 5.8% | 65 | 6.7% | 19 | 7.5% | 10 | 4.7% | 2 | 1.3% | 6 | 3.8% |
| Self-efficacy to ask patients if they hear voices | 98 | 5.6% | 60 | 6.2% | 20 | 7.9% | 10 | 4.7% | 2 | 1.3% | 6 | 3.8% |
| Self-efficacy to discuss voice-hearing | 94 | 5.4% | 57 | 5.9% | 19 | 7.5% | 10 | 4.7% | 2 | 1.3% | 6 | 3.8% |
| Training in helping service users with distressing voice-hearing | 34 | 1.9% | 22 | 2.3% | 6 | 2.4% | 2 | 0.9% | 2 | 1.3% | 2 | 1.3% |
| Personal Experience with voice-hearing | 15 | 0.9% | 10 | 1.0% | 3 | 1.2% | 1 | 0.5% | 0 | - | 1 | 0.6% |
| The AAPPQ subscales and AQ-9, the descriptive statistics presented are based on the reduced scales that were used in the study analyses. TPB = Theory of Planned Behavior; m-AAPPQ = modified Alcohol and Alcohol Problems Perception Questionnaire; AQ-9 = Attribution Questionnaire-9. | | | | | | | | | | | | |

**Table S4.** *Missing cases and percentages in the indirect TPB belief items in EIP and CAMHS clinicians.*

|  | EIP (*N* = 253) | | CAMHS  (*N* = 214) | |
| --- | --- | --- | --- | --- |
|  | *N* | % | *N* | % |
| **Control Beliefs** |  |  |  |  |
| I have had training or knowledge in assessing voice-hearing in young people | 59 | 23.3% | 66 | 30.8% |
| young people who hear distressing voices present with high-risk issues. | 59 | 23.3% | 66 | 30.8% |
| young people present with practical issues (e.g., lack of permanent address, current life stressors) that seem more immediate than their voice-hearing experiences. | 58 | 22.9% | 65 | 30.4% |
| I have access to adequate collateral information (e.g., family history, young person’s culture, values) about the young people I am working with. | 57 | 22.5% | 65 | 30.4% |
| young people who hear distressing voices do not have the ability to answer assessment questions relating to their voice-hearing experiences. | 57 | 22.5% | 62 | 29.0% |
| I have the opportunity to consult my team on how to proceed with my cases. | 57 | 22.5% | 63 | 29.4% |
| people in the social system of young people (e.g., carers, friends) are accepting of the young person’s voice-hearing experiences. | 57 | 22.5% | 62 | 29.0% |
| young people who hear distressing voices are very unwell to engage in an assessment of their voice-hearing experiences. | 57 | 22.5% | 64 | 29.9% |
| voice-hearing assessment tools (e.g., assessment measures, questionnaires) are available to me. | 56 | 22.1% | 62 | 29.0% |
| young people who disclose hearing distressing voices to me are unwilling to discuss their voice-hearing experiences with me. | 55 | 21.7% | 62 | 29.0% |
| I have good engagement with young people who hear distressing voices. | 55 | 21.7% | 61 | 28.5% |
| I can find a suitable space to assess young people’s experiences. | 55 | 21.7% | 61 | 28.5% |
| I have limited time to assess young people’s experiences. | 55 | 21.7% | 61 | 28.5% |
| **Subjective Norm beliefs** |  |  |  |  |
| clinicians in the same profession as me would assess the young person's voice-hearing experiences | 52 | 20.6% | 59 | 27.6% |
| other colleagues in my clinical team would assess the young person’s voice-hearing experiences. | 51 | 20.2% | 58 | 27.1% |
| individuals in the young person’s social system (e.g., family, friends) think I should assess the young person’s voice-hearing experiences. | 50 | 19.8% | 56 | 26.2% |
| specialist mental health practitioners (e.g., psychologists, psychiatrists) think I should assess the young person’s voice-hearing experiences | 50 | 19.8% | 56 | 26.2% |
| the young person thinks I should assess their voice-hearing experiences | 48 | 19.0% | 56 | 26.2% |
| **Behavioral beliefs** |  |  |  |  |
| would help evaluate the impact of voices on the young person’s emotions (e.g., distress). | 45 | 17.8% | 49 | 22.9% |
| would help evaluate the impact of voices on the young person’s functioning | 44 | 17.4% | 49 | 22.9% |
| would lead to mistakenly labelling the young person with a mental health disorder such as psychosis | 44 | 17.4% | 52 | 24.3% |
| would help with assessing the risk to self/others that is related to voices. | 44 | 17.4% | 48 | 22.4% |
| would make the young person feel distressed (e.g., anxious, fearful). | 44 | 17.4% | 51 | 23.8% |
| would aid diagnosing. | 44 | 17.4% | 51 | 23.8% |
| would lead to over-focusing on voices and incomplete exploration of other critical areas of a young person’s presentation. | 44 | 17.4% | 50 | 23.4% |
| would put engagement with the young person at risk. | 44 | 17.4% | 51 | 23.8% |
| would be reassuring and validating for the young person | 42 | 16.6% | 45 | 21.0% |
| would help promote good engagement between me and the young person | 42 | 16.6% | 42 | 19.6% |
| would help identify the right support or treatment if needed. | 42 | 16.6% | 42 | 19.6% |
| would help with constructing a detailed formulation of what is happening for the young person. | 42 | 16.6% | 42 | 19.6% |

**Table S5.** *Descriptive statistics on all variables of interest by service group, including both young people and adult versions of the A2V survey (N = 1751).*

| Variable | Adult Mental health  (*N* = 996) | | | EIP (*N* = 253) | | | CAMHS (*N* = 214) | | | Primary Care (Adult patient version)  (*N* = 158) | | | Primary Care  (Young people version) (*N* = 160) | | | All participants  (*N* = 1751) | | | |
| --- | --- | --- | --- | --- | --- | --- | --- | --- | --- | --- | --- | --- | --- | --- | --- | --- | --- | --- | --- |
|  | *N* | *M* (Min-Max) | *SD* | *N* | *M* (Min-Max) | *SD* | *N* | *M* (Min-Max) | *SD* | *N* | *M* (Min-Max) | *SD* | *N* | *M* (Min-Max) | *SD* | *N* | *M* (Min-Max) | *SD* |  |
| Self-efficacy to ask patients if they hear voices | 906 | 91.00 (0-100) | 16.38 | 233 | 95.67 (20-100) | 9.69 | 204 | 89.77 (14-100) | 17.37 | 156 | 73.57 (0-100) | 30.15 | 154 | 70.72 (0-100) | 29.86 | 1653 | 87.97 (0-100) | 20.64 |  |
| Self-efficacy to discuss voice-hearing | 909 | 88.03 (4-100) | 17.55 | 234 | 93.63 (10-100) | 11.48 | 204 | 84.67 (11-100) | 20.94 | 156 | 64.87 (0-100) | 31.59 | 154 | 62.86 (0-100) | 30.13 | 1657 | 83.89 (0-100) | 22.73 |  |
| Self-efficacy to provide useful information | 901 | 69.38 (0-100) | 26.74 | 234 | 81.57 (20-100) | 19.52 | 204 | 63.54 (0-100) | 26.61 | 156 | 32.69 (0-100) | 26.43 | 154 | 35.71 (0-100) | 27.98 | 1649 | 63.77 (0-100) | 29.95 |  |
| Self-efficacy to refer | - | - | - | - | - | - | - | - | - | 156 | 73.74 (0-100) | 30.32 | 154 | 70.98 (0-100) | 33.11 | 310 | 72.37 (0-100) | 31.71 |  |
| TPB attitudes | 825 | 5.43 (1.86- 7) | .93 | 212 | 5.79 (1.57-7) | .85 | 176 | 5.35 (2.14-7) | .89 | 150 | 4.72 (1-6.86) | 1.16 | 143 | 4.55 (1-6.71) | 1.12 | 1506 | 5.32 (1-7) | 1.02 |  |
| TPB subjective norms | 853 | 5.38 (1.2-7) | 1.06 | 221 | 5.80 (2.20 – 7) | .92 | 185 | 5.47 (1.8-7) | 1.03 | 150 | 4.66 (1.2-7) | 1.37 | 146 | 4.67 (1.4-7) | 1.27 | 1555 | 5.32 (1.2-7) | 1.14 |  |
| TPB perceived behavioral control | 823 | 5.14 (1-7) | 1.07 | 212 | 5.51 (1.17-7) | .87 | 174 | 5.03 (2-7) | 1.14 | 150 | 4.49 (1-6.50) | 1.24 | 143 | 4.39 (1.17- 6.67) | 1.16 | 1502 | 5.04 (1-7) | 1.13 |  |
| TPB intention | 818 | 5.76 (1-7) | 1.23 | 194 | 6.33 (2.33-7) | .91 | 148 | 6.04 (2-7) | 1.05 | 149 | 5.21 (1-7) | 1.46 | 141 | 5.11 (1-7) | 1.66 | 1450 | 5.75 (1-7) | 1.30 |  |
| m-AAPPQ Therapeutic Commitment | 789 | 5.51 (2.51-7) | .74 | 190 | 5.87 (1.93- 7) | .71 | 144 | 5.27 (2.77-7) | .74 | 145 | 4.46 (2.27- 6.03) | .70 | 138 | 4.34 (2.21-6.71) | .78 | 1406 | 5.31 (1.93-7) | .88 |  |
| m-AAPPQ Role security | 800 | 5.12 (1.75 -7 ) | .91 | 189 | 5.54 (1.88-7) | .80 | 144 | 4.97 (2.33-7) | .91 | 146 | 4.27 (1-6.75) | .98 | 138 | 4.04 (1.75-6.92) | 1.06 | 1417 | 4.97 (1-7) | 1.01 |  |
| m-AAPPQ Empathy | 794 | 4.97 (1-7) | 1.03 | 189 | 5.33 (1.75-7) | .92 | 144 | 4.94 (2-7) | .98 | 145 | 4.37 (2-6.25) | .95 | 138 | 4.55 (2.5-6.75) | .97 | 1410 | 4.92 (1-7) | 1.03 |  |
| AQ-9 Stigma | 786 | 19.41 (9-81) | 7.68 | 189 | 19.19 (9-39) | 6.53 | 141 | 18.87 (9-39) | 6.31 | 144 | 24.19 (9-49) | 8.24 | 137 | 24.36 (9-54) | 7.49 | 1397 | 20.30 (9-81) | 7.70 |  |
|  |  | ***N* (%)** |  |  | ***N* (%)** |  |  | ***N* (%)** |  |  | ***N* (%)** |  |  | ***N* (%)** |  |  | ***N* (%)** |  |  |
| Personal Experience with voice-hearing | 956 |  |  | 250 |  |  | 213 |  |  | 158 |  |  | 159 |  |  | 1736 |  |  |  |
| Yes |  | 264 (27.62) |  |  | 91 (36.40) |  |  | 66 (30.99) |  |  | 25 (15.82) |  |  | 35 (22.01) |  |  | 481 (27.71) |  |  |
| No |  | 692 (72.38) |  |  | 159 (63.60) |  |  | 147 (69.01) |  |  | 133 (84.18) |  |  | 124 (77.99) |  |  | 1255 (72.29) |  |  |
| Training in helping service users with distressing voice-hearing | 944 |  |  | 247 |  |  | 212 |  |  | 156 |  |  | 158 |  |  | 1717 |  |  |  |
| Formal training |  | 507 (53.71) |  |  | 175 (70.85) |  |  | 95 (44.81) |  |  | 39 (25) |  |  | 34 (21.52) |  |  | 850 (49.50) |  |  |
| No formal training/Considerable clinical experience |  | 365 (38.67) |  |  | 65 (26.32) |  |  | 71 (33.49) |  |  | 47 (30.13) |  |  | 38 (24.05) |  |  | 586 (34.13) |  |  |
| No formal training/No or limited clinical experience |  | 72 (7.63) |  |  | 7 (2.83) |  |  | 46 (21.70) |  |  | 70 (44.87) |  |  | 86 (54.43) |  |  | 281 (16.37) |  |  |
| Professional experience working with voice-hearers | 966 |  |  | 253 |  |  | 149 |  |  | 158 |  |  | 160 |  |  | 1751 |  |  |  |
| No experience |  | 14 (1.45) |  |  | 1 (.40) |  |  | 8 (3.74) |  |  | 22 (13.92) |  |  | 29 (18.13) |  |  | 74 (4.23) |  |  |
| 1-2 service users |  | 37 (3.83) |  |  | 2 (.79) |  |  | 25 (11.68) |  |  | 18 (11.39) |  |  | 17 (10.63) |  |  | 99 (5.65) |  |  |
| 3-4 service users |  | 44 (4.55) |  |  | 14 (5.53) |  |  | 16 (7.48) |  |  | 16 (10.13) |  |  | 18 (11.25) |  |  | 108 (6.17) |  |  |
| 5-9 service users |  | 96 (9.94) |  |  | 23 (9.09) |  |  | 16 (7.48) |  |  | 26 (16.46) |  |  | 23 (14.37) |  |  | 184 (10.51) |  |  |
| 10+ service users |  | 775 (80.23) |  |  | 213 (84.19) |  |  | 149 (69.63) |  |  | 76 (48.10) |  |  | 73 (45.63) |  |  | 1286 (73.44) |  |  |
| Frequency of contact with voice-hearers in clinical practice | 938 |  |  | 230 |  |  | 196 |  |  | 127 |  |  | 99 |  |  | 1590 |  |  |  |
| Less than once a month |  | 123 (13.11) |  |  | 79 (34.35) |  |  | 74 (37.76) |  |  | 109 (68.99) |  |  | 90 (90.91) |  |  | 475 (29.87) |  |  |
| 1-3 times a month |  | 190 (20.26) |  |  | 70 (30.34) |  |  | 57 (29.08) |  |  | 13 (10.24) |  |  | 6 (6.96) |  |  | 336 (21.13) |  |  |
| Once a week |  | 145 (15.46) |  |  | 35 (15.22) |  |  | 35 (17.86) |  |  | 0 |  |  | 2 (2.02) |  |  | 217 (13.65) |  |  |
| A few times a week |  | 240 (25.59) |  |  | 34 (14.78) |  |  | 15 (7.65) |  |  | 4 (3.15) |  |  | 1 (1.01) |  |  | 294 (18.49) |  |  |
| Everyday |  | 181 (19.30) |  |  | 11 (4.78) |  |  | 12 (6.12) |  |  | 1 (.79) |  |  | 0 |  |  | 205 (12.89) |  |  |
| More than once a day |  | 59 (6.29) |  |  | 1 (.43) |  |  | 3 (1.53) |  |  | 0 |  |  | 0 |  |  | 63 (3.96) |  |  |
| The AAPPQ subscales and AQ-9, the descriptive statistics presented are based on the full scales before item removal to improve their internal consistency. TPB = Theory of Planned Behaviour; m-AAPPQ = modified Alcohol and Alcohol Problems Perception Questionnaire; AQ-9 = Attribution Questionnaire-9. | | | | | | | | | | | | | | | | | | | |

**Table S6.** *Descriptive statistics for additional clinically relevant variables by service group (N = 1751)*

| Variable | Adult Mental health  (*N* = 996) | | | EIP (*N* = 253) | | | CAMHS (*N* = 214) | | | Primary Care (Adult patient version)  (*N* = 158) | | | Primary Care  (Young people version)  (*N* = 160) | | | All participants  (*N* = 1751) | | |
| --- | --- | --- | --- | --- | --- | --- | --- | --- | --- | --- | --- | --- | --- | --- | --- | --- | --- | --- |
|  | *N* | *M* (Min-Max) | *SD* | *N* | *M* (Min-Max) | *SD* | *N* | *M* (Min-Max) | *SD* | *N* | *M* (Min-Max) | *SD* | *N* | *M* (Min-Max) | *SD* | *N* | *M* (Min-Max) | *SD* |
|  |  |  |  |  |  |  |  |  |  |  |  |  |  |  |  |  |  |  |
| m-AAPPQ Difficulty working with voice-hearers | 793 | 4.77 (0-10) | 2.36 | 190 | 4.48 (0-10) | 2.45 | 144 | 4.81 (0-10) | 2.25 | 145 | 5.57 (0-10) | 2.09 | 138 | 6.31 (0-10) | 2.19 | 1410 | 4.97 (0-10) | 2.37 |
|  |  | ***N*(Valid %)** |  |  | **N (Valid %)** |  |  | **N (Valid %)** |  |  | **N (Valid %)** |  |  | **N (Valid %)** |  |  | **N (Valid %)** |  |
| Perceived need for training to support voice-hearers | 913 |  |  | 234 |  |  | 205 |  |  | 156 |  |  | 154 |  |  | 1662 |  |  |
| Yes |  | 610 (66.81) |  |  | 145 (61.97) |  |  | 154 (75.12) |  |  | 99 (63.46) |  |  | 92 (59.74) |  |  | 1100 (66.19) |  |
| No |  | 138 (15.12) |  |  | 34 914.53) |  |  | 21 (10.24) |  |  | 25 (16.03) |  |  | 23 (14.94) |  |  | 241 (14.50) |  |
| Not sure |  | 165 (18.07) |  |  | 55 (23.50) |  |  | 30 (14.63) |  |  | 32 (20.51) |  |  | 39 (25.32) |  |  | 321 (19.31) |  |
| Perceived need for training to assess voice-hearing | 911 |  |  | 234 |  |  | 204 |  |  | 156 |  |  | 154 |  |  | 1659 |  |  |
| Yes |  | 459 (50.38) |  |  | 110 (47.01) |  |  | 127 (62.25) |  |  | 77 (49.36) |  |  | 80 (51.95) |  |  | 853 (51.42) |  |
| No |  | 278 (30.52) |  |  | 76 (32.48) |  |  | 44 (21.57) |  |  | 40 (25.64) |  |  | 30 (19.48) |  |  | 468 (28.21) |  |
| Not sure |  | 174 (19.10) |  |  | 48 (20.51) |  |  | 33 (16.18) |  |  | 39 (25) |  |  | 44 (28.57) |  |  | 338 (20.37) |  |
| Willingness to receive training in supporting voice-hearers | 911 |  |  | 234 |  |  | 205 |  |  | 156 |  |  | 154 |  |  | 1660 |  |  |
| Yes |  | 878 (96.38) |  |  | 226 (96.58) |  |  | 198 (96.59) |  |  | 137 (87.82) |  |  | 129 (83.77) |  |  | 1568 (94.46) |  |
| No |  | 10 (1.10) |  |  | 2 (.85) |  |  | 2 (.98) |  |  | 8 (5.13) |  |  | 6 (3.90) |  |  | 28 (1.690 |  |
| Not sure |  | 23 (2.52) |  |  | 6 (2.56) |  |  | 5 (2.44) |  |  | 11 (7.05) |  |  | 19 (12.34) |  |  | 64 (3.86) |  |
| Willingness to receive training in assessing voice-hearing | 911 |  |  | 234 |  |  | 204 |  |  | 156 |  |  | 154 |  |  | 1659 |  |  |
| Yes |  | 832 (91.33) |  |  | 219 (93.59) |  |  | 192 (94.12) |  |  | 130 (83.33) |  |  | 130 (84.42) |  |  | 1503 (90.60) |  |
| No |  | 40 (4.39) |  |  | 7 (2.99) |  |  | 4 (1.96) |  |  | 8 (5.13) |  |  | 8 (5.19) |  |  | 67 (4.04) |  |
| Not sure |  | 39 (4.28) |  |  | 8 (3.42) |  |  | 8 (3.92) |  |  | 18 (11.54) |  |  | 16 (10.39) |  |  | 89 (5.36) |  |
| m-AAPPQ = modified Alcohol and Alcohol Problems Perception Questionnaire. | | | | | | | | | | | | | | | | | | |

**Table S7**. *Between-group differences in weighted belief items comparing the no/low (N = 32) vs. the medium/high intention (N = 116) to assess voice-hearing groups in CAMHS clinicians.*

|  | No/Low intention group | | | Medium/high intention group | | |  |  |  |  |
| --- | --- | --- | --- | --- | --- | --- | --- | --- | --- | --- |
| **Weighted belief item** | *N* | *Mean* | *SD* | *N* | *Mean* | *SD* | *t* (df) | *d* | p | BCa 95% of Mean Dif [LL, UL] |
| **Behavioural beliefs** |  |  |  |  |  |  |  |  |  |  |
| ***Assessing voice-hearing…*** |  |  |  |  |  |  |  |  |  |  |
| would help with constructing a detailed formulation of what is happening for the young person. | 31 | 18.19 | 2.56 | 116 | 19.66 | 1.87 | -2.97 (38.95) | 0.66 | 0.004 | [-2.44, -.60] |
| would put engagement with the young person at risk. | 32 | -3.53 | 5.63 | 116 | -3.91 | 4.05 | .35 (40.29) | -0.08 | 0.73 | [-1.62, 2.74] |
| would help identify the right support or treatment if needed. | 32 | 17.81 | 2.84 | 116 | 19.11 | 2.19 | -2.40 (41.62) | 0.51 | 0.015 | [-2.40, -.26] |
| would help promote good engagement between me and the young person | 32 | 16.78 | 3.13 | 116 | 18.36 | 2.17 | -2.69 (39.61) | 0.59 | 0.018 | [-2.79, -.43] |
| would lead to over-focusing on voices and incomplete exploration of other critical areas of a young person’s presentation. | 32 | -5.81 | 3.94 | 116 | -5.91 | 4.05 | .12 (50.60) | -0.03 | 0.923 | [-1.62, 1.55] |
| would aid diagnosing. | 32 | 0.84 | 4.87 | 116 | 2.36 | 7.38 | -1.38 (74.57) | 0.24 | 0.169 | [-3.67, .66] |
| would be reassuring and validating for the young person | 32 | 16.69 | 2.95 | 116 | 18.26 | 2.52 | -2.75 (44.30) | 0.57 | 0.011 | [-2.77, -.46] |
| would make the young person feel distressed (e.g., anxious, fearful). | 32 | -6.78 | 4.5 | 116 | -4.84 | 4.83 | -2.12 (52.42) | 0.42 | 0.045 | [-3.70, -.11] |
| would help with assessing the risk to self/others that is related to voices. | 32 | 11.75 | 1.41 | 116 | 12.59 | 1.29 | -3.02 (46.27) | 0.62 | 0.008 | [-1.40, -.26] |
| would lead to mistakenly labelling the young person with a mental health disorder such as psychosis | 32 | -8.78 | 4.35 | 116 | -6.07 | 3.68 | -3.23 (44.01) | 0.67 | 0.003 | [-4.34, -1.08] |
| would help evaluate the impact of voices on the young person’s functioning | 32 | 17.44 | 2.58 | 116 | 18.91 | 2.66 | -2.83 (50.73) | 0.56 | 0.005 | [-2.51, -.43] |
| would help evaluate the impact of voices on the young person’s emotions (e.g., distress). | 32 | 18 | 2.29 | 116 | 19.27 | 2.02 | -2.84 (45.27) | 0.59 | 0.013 | [-2.14, -.41] |
| **Normative beliefs** |  |  |  |  |  |  |  |  |  |  |
| the young person thinks I should assess their voice-hearing experiences | 32 | 0.38 | 5.92 | 116 | 5.85 | 6.49 | -4.54 (53.37) | 0.88 | **0.001** | [-7.96, -3.20] |
| specialist mental health practitioners (e.g., psychologists, psychiatrists) think I should assess the young person’s voice-hearing experiences | 32 | -2.91 | 9 | 116 | 9.26 | 6.7 | -7.12 (40.93) | 1.53 | **0.001** | [-15.62, 8.82] |
| individuals in the young person’s social system (e.g., family, friends) think I should assess the young person’s voice-hearing experiences. | 32 | 3.66 | 5.4 | 116 | 8.94 | 5.88 | -4.80 (53.00) | 0.94 | **0.001** | [-7.50, -3.13] |
| other colleagues in my clinical team would assess the young person’s voice-hearing experiences. | 32 | 6.66 | 5.56 | 116 | 8.29 | 6.33 | -1.43 (55.28) | 0.27 | 0.17 | [-3.87, .87] |
| clinicians in the same profession as me would assess the young person's voice-hearing experiences | 32 | 1.22 | 7.03 | 116 | 9.76 | 6.16 | -6.24 (44.98) | 1.29 | **0.001** | [-11.46, -5.72] |
| **Control beliefs** |  |  |  |  |  |  |  |  |  |  |
| I have limited time to assess young people’s experiences. | 32 | -7.78 | 5.77 | 116 | -4.47 | 5.61 | -2.89 (48.33) | 0.58 | 0.008 | [-5.45, -.83] |
| I can find a suitable space to assess young people’s experiences. | 32 | 7.09 | 7.28 | 116 | 9.26 | 8.28 | -1.44 (55.13) | 0.28 | 0.17 | [-4.99, 1.17] |
| young people who hear distressing voices are very unwell to engage in an assessment of their voice-hearing experiences. | 32 | -5.88 | 5.2 | 116 | -2.97 | 4.14 | -2.91 (42.42) | 0.62 | 0.006 | [-4.76, .95] |
| I have good engagement with young people who hear distressing voices. | 32 | 10.63 | 4.45 | 116 | 11.91 | 6.02 | -1.34 (65.69) | 0.24 | 0.15 | [-3.12, .60] |
| young people present with practical issues (e.g., lack of permanent address, current life stressors) that seem more immediate than their voice-hearing experiences. | 32 | -3.69 | 7.19 | 116 | -1.84 | 6.13 | -1.32 (44.20) | 0.28 | 0.192 | [-4.53, .72] |
| young people who disclose hearing distressing voices to me are unwilling to discuss their voice-hearing experiences with me. | 32 | -4.94 | 5.47 | 116 | -2.7 | 4.03 | -2.16 (40.71) | 0.47 | 0.052 | [-4.46, -.24] |
| young people who hear distressing voices present with high-risk issues. | 32 | -2.44 | 6.2 | 116 | -0.26 | 7.41 | -1.69 (57.88) | 0.32 | 0.092 | [-4.85, .20] |
| voice-hearing assessment tools (e.g., assessment measures, questionnaires) are available to me. | 32 | 3.88 | 6.88 | 116 | 5.85 | 5.36 | -1.51 (41,94) | 0.32 | 0.145 | [-4.82, .44] |
| people in the social system of young people (e.g., carers, friends) are accepting of the young person’s voice-hearing experiences. | 32 | 4.38 | 5.53 | 116 | 4.53 | 3.8 | -.145 (39.39) | 0.03 | 0.863 | [-2.06, 1.89] |
| I have the opportunity to consult my team on how to proceed with my cases. | 32 | 11.5 | 7.84 | 116 | 11.37 | 7.24 | .084 (46.59) | -0.02 | 0.933 | [-2.97, 3.23] |
| young people who hear distressing voices do not have the ability to answer assessment questions relating to their voice-hearing experiences. | 32 | -4.16 | 4.16 | 116 | -2.73 | 3.86 | -1.74 (46.78) | 0.36 | 0.075 | [-3.02, .08] |
| I have access to adequate collateral information (e.g., family history, young person’s culture, values) about the young people I am working with. | 32 | 8.84 | 7.24 | 116 | 8.99 | 5.75 | -.11 (42.37) | 0.02 | 0.92 | [-3.07, 2.48] |
| I have had training or knowledge in assessing voice-hearing in young people | 32 | 5.13 | 4.05 | 116 | 8.63 | 6.25 | -3.81 (76.28) | 0.66 | **0.001** | [-5.35, -1.65] |
| *SD* = standard deviation; *d f* = degrees of freedom; *d* = Cohen’s d; BCa 95% of Mean Difference and p-values are based on bootstrapping with *N =* 2000 samples; LL = lower limit; UL = upper limit; All tests were run with untransformed variables. Significance values are presented uncorrected for multiple comparisons. Bonferroni corrected p-value for the t-tests is .002. Significance values in bold font highlight the statistically significant effects. | | | | | | | | | | |

**Table S8.** *Between-group differences in weighted belief items comparing the no/low (N = 32) vs. the medium/high intention (N = 163) to assess voice-hearing groups in EIP clinicians.*

|  | No/Low intention group | | | Medium/high intention group | | |  |  |  |  |
| --- | --- | --- | --- | --- | --- | --- | --- | --- | --- | --- |
| **Weighted belief item** | *N* | *Mean* | *SD* | *N* | *Mean* | *SD* | *t* (*df*) | *d* | *p* | BCa 95% of Mean Dif [LL, UL] |
| **Behavioral beliefs** |  |  |  |  |  |  |  |  |  |  |
| ***Assessing voice-hearing…*** |  |  |  |  |  |  |  |  |  |  |
| would help with constructing a detailed formulation of what is happening for the young person. | 31 | 17.32 | 3.35 | 163 | 20.06 | 1.58 | -4.46 (32.59) | 1.05 | **0.001** | [-3.95, -1.53] |
| would put engagement with the young person at risk. | 31 | -6.26 | 6.22 | 163 | -3.84 | 4.34 | -2.07 (35.76) | 0.45 | 0.048 | [-4.78, -.21] |
| would help identify the right support or treatment if needed. | 31 | 16.84 | 3.77 | 163 | 19.47 | 2.01 | -3.79 (33.32) | 0.87 | **0.001** | [-4.05, -1.33] |
| would help promote good engagement between me and the young person | 31 | 15.77 | 2.89 | 163 | 18.15 | 2.86 | -4.19 (41.94) | 0.83 | **0.001** | [-3.43, -1.19] |
| would lead to over-focusing on voices and incomplete exploration of other critical areas of a young person’s presentation. | 31 | -7.29 | 5.46 | 163 | -4.26 | 3.41 | -2.98 (34.59) | 0.67 | 0.007 | [-5.05, -1.04] |
| would aid diagnosing. | 31 | -1.32 | 7.85 | 163 | 0.58 | 7.94 | -1.23 (42.52) | 0.24 | 0.211 | [-4.63, 1.05] |
| would be reassuring and validating for the young person | 31 | 15.87 | 3.39 | 163 | 17.91 | 2.48 | -3.18 (36.34) | 0.69 | 0.004 | [-3.21, -.75] |
| would make the young person feel distressed (e.g., anxious, fearful). | 31 | -8.13 | 5.28 | 163 | -6.34 | 5.24 | -1.73 (42.05) | 0.34 | 0.079 | [-4.00, -.07] |
| would help with assessing the risk to self/others that is related to voices. | 31 | 11.29 | 1.68 | 162 | 12.6 | 1.46 | -4.08 (39.22) | 0.83 | **0.001** | [-1.91, -.65] |
| would lead to mistakenly labelling the young person with a mental health disorder such as psychosis. | 31 | -8.48 | 5.7 | 163 | -5.98 | 3.96 | -2.34 (35.70) | 0.51 | 0.021 | [-4.68, -.46] |
| would help evaluate the impact of voices on the young person’s functioning. | 31 | 17.32 | 2.76 | 163 | 19.64 | 1.64 | -4.52 (34.13) | 1.02 | **0.001** | [-3.29, -1.32] |
| would help evaluate the impact of voices on the young person’s emotions (e.g., distress). | 31 | 17.81 | 2.89 | 163 | 19.66 | 1.57 | -3.47 (33.43) | 0.80 | 0.003 | [-3.01, -.85] |
| **Normative beliefs** |  |  |  |  |  |  |  |  |  |  |
| the young person thinks I should assess their voice-hearing experiences | 31 | 2.81 | 5.23 | 163 | 5.68 | 6.57 | -2.68 (49.89) | 0.48 | 0.007 | [-4.86, -.99] |
| specialist mental health practitioners (e.g., psychologists, psychiatrists) think I should assess the young person’s voice-hearing experiences | 31 | -1.42 | 9.57 | 163 | 11.77 | 5.19 | -7.47 (33.44) | 1.71 | **0.001** | [-16.60, -9.73] |
| individuals in the young person’s social system (e.g., family, friends) think I should assess the young person’s voice-hearing experiences. | 31 | 4.35 | 5.29 | 163 | 9.09 | 4.76 | -4.64 (39.78) | 0.94 | **0.001** | [-6.72, -2.60] |
| other colleagues in my clinical team would assess the young person’s voice-hearing experiences. | 31 | 8.65 | 6.37 | 163 | 10.25 | 6.16 | -1.29 (41.38) | 0.26 | 0.211 | [-4.18, .84] |
| clinicians in the same profession as me would assess the young person's voice-hearing experiences | 31 | 1.77 | 9.42 | 163 | 10.8 | 6.65 | -5.09 (35.93) | 1.11 | **0.001** | [-12.62, -5.83] |
| **Control beliefs** |  |  |  |  |  |  |  |  |  |  |
| I have limited time to assess young people’s experiences. | 31 | -4.74 | 6.48 | 163 | -3.59 | 6.26 | -.91 (41.36) | 0.18 | 0.388 | [-3.68, 1.54] |
| I can find a suitable space to assess young people’s experiences. | 31 | 4.61 | 7.44 | 163 | 10.57 | 7.13 | -4.12 (41.12) | 0.82 | **0.001** | [-9.08, -3.18] |
| young people who hear distressing voices are very unwell to engage in an assessment of their voice-hearing experiences. | 31 | -4.32 | 5.54 | 163 | -3.07 | 4.03 | -1.20 (36.26) | 0.26 | 0.228 | [-3.32, .71] |
| I have good engagement with young people who hear distressing voices. | 31 | 10.19 | 7.67 | 163 | 13.51 | 4.82 | -2.32 (34.65) | 0.52 | 0.032 | [-6.14, -.70] |
| young people present with practical issues (e.g., lack of permanent address, current life stressors) that seem more immediate than their voice-hearing experiences. | 31 | -5.19 | 6.72 | 163 | -1.73 | 6.8 | -2.63 (42.55) | 0.51 | 0.013 | [-6.23, -1.08] |
| young people who disclose hearing distressing voices to me are unwilling to discuss their voice-hearing experiences with me. | 31 | -4.45 | 5.2 | 163 | -3.67 | 3.8 | -.80 (36.36) | 0.17 | 0.429 | [-2.66, 1.29] |
| young people who hear distressing voices present with high-risk issues. | 31 | -2.61 | 6.81 | 163 | 0.82 | 6.7 | -2.58 (41.80) | 0.51 | 0.014 | [-6.03, -.78] |
| voice-hearing assessment tools (e.g., assessment measures, questionnaires) are available to me. | 31 | 6.65 | 5.15 | 163 | 9.34 | 7.02 | -2.51 (53.74) | 0.44 | 0.019 | [-4.75, -.52] |
| people in the social system of young people (e.g., carers, friends) are accepting of the young person’s voice-hearing experiences. | 31 | 4.61 | 3.94 | 163 | 5.1 | 5.07 | -.61 (50.99) | 0.11 | 0.564 | [-2.02, 1.19] |
| I have the opportunity to consult my team on how to proceed with my cases. | 31 | 11.1 | 6.28 | 163 | 11.33 | 7.36 | -.19 (47.09) | 0.03 | 0.851 | [-2.80, 2.14] |
| young people who hear distressing voices do not have the ability to answer assessment questions relating to their voice-hearing experiences. | 31 | -3.32 | 3.03 | 163 | -2.52 | 3.93 | -1.28 (51.30) | 0.23 | 0.207 | [-2.09, .36] |
| I have access to adequate collateral information (e.g., family history, young person’s culture, values) about the young people I am working with. | 31 | 7.16 | 6.07 | 163 | 8.6 | 6.14 | -1.21 (42.49) | 0.24 | 0.248 | [-3.86, .95] |
| I have had training or knowledge in assessing voice-hearing in young people | 31 | 5.71 | 4.93 | 163 | 11.07 | 6.35 | -5.28 (51.01) | 0.94 | **0.001** | [-7.39, -3.34] |
| *SD* = standard deviation; *df* = degrees of freedom; *d* = Cohen’s d; BCa 95% of Mean Difference and p-values are based on bootstrapping with *N =* 2000 samples; LL = lower limit; UL = upper limit; All tests were run with untransformed variables. Significance values are presented uncorrected for multiple comparisons. Bonferroni corrected p-value for the t-tests is .002. Significance values in bold font highlight the statistically significant effects. | | | | | | | | | | |
